# A thalamic hub-and-spoke network enables visual perception during action by coordinating visuomotor dynamics

**DOI:** 10.1038/s41593-025-01874-w

**Published:** 2025-02-10

**Authors:** Tomas Vega-Zuniga, Anton Sumser, Olga Symonova, Peter Koppensteiner, Florian H. Schmidt, Maximilian Joesch

**Affiliations:** 1 Institute of Science and Technology Austria, Klosterneuburg, Austria; 2 Present Address: Division of Neuroscience, Faculty of Biology, LMU Munich, Martinsried, Germany

**Keywords:** Thalamus, Sensorimotor processing, Motor control, Neural circuits

## Abstract

For accurate perception and motor control, an animal must distinguish between sensory experiences elicited by external stimuli and those elicited by its own actions. The diversity of behaviors and their complex influences on the senses make this distinction challenging. Here, we uncover an action–cue hub that coordinates motor commands with visual processing in the brain’s first visual relay. We show that the ventral lateral geniculate nucleus (vLGN) acts as a corollary discharge center, integrating visual translational optic flow signals with motor copies from saccades, locomotion and pupil dynamics. The vLGN relays these signals to correct action-specific visual distortions and to refine perception, as shown for the superior colliculus and in a depth-estimation task. Simultaneously, brain-wide vLGN projections drive corrective actions necessary for accurate visuomotor control. Our results reveal an extended corollary discharge architecture that refines early visual transformations and coordinates actions via a distributed hub-and-spoke network to enable visual perception during action.

## Abstract (web summary)

Animals must distinguish between signals from sensory stimuli and action-generated signals. This paper shows that the ventral lateral geniculate nucleus acts as a corollary discharge center to suppress motion-induced visual signals, orchestrating accurate perception and motor control.

## Main

**Fig. 1:**
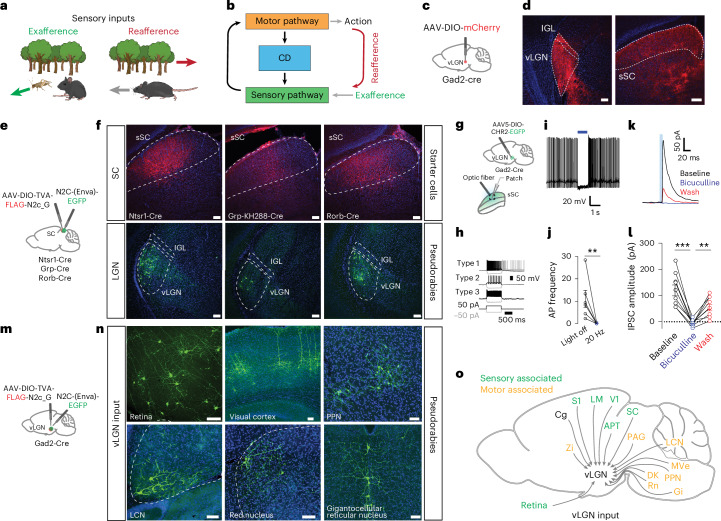
Structural and functional evidence of a thalamic CD pathway. **a**, Schematic showing the difference between external visual input (exafference, green arrow) and visual input generated by the animal’s own motion (reafference, red arrow). **b**, Schematic showing a sensorimotor circuit with a motor-copy signal, known as a CD (blue), bridging a sensory (green) and a motor pathway (orange). **c**, Brain schematic showing the location of anterograde vector injection in the vLGN (red dot). **d**, Injection site in vLGN (left) and expression of mCherry vector in SC (right, *n* = 3 animals). **e**, Schematic showing the location of retrograde transsynaptic vector injections in the sSC. **f**, Coronal brain sections of the three transgenic lines. Top, sSC with starter vector expression in red. Bottom, transsynaptic EGFP expression by the pseudorabies vectors in the vLGN (*n* = 6 animals). **g**, Schematic brain showing a Gad2^+^ vLGN injection of ChR2 and subsequent patch-clamp recordings in the sSC. **h**, Intrinsic firing properties of sSC cell types in response to current steps. **i**, Example current-clamp recording(s) of spontaneous activity of sSC cell(s) suppressed by 20-Hz optogenetic stimulation. **j**, Quantification of optogenetic suppression of sSC neurons as in **i**. (6 cells, 3 animals, Mann–Whitney *U* two-sided test, *P* = 0.0037, data are presented as mean ± s.e.m.). **k**, Example voltage-clamp recording of an sSC neuron in the presence of bicuculline and subsequent wash. Inhibitory currents induced by optogenetics are reversibly blocked by bicuculline (20 µM). **l**, Inhibitory postsynaptic current (IPSC) amplitude quantification of all recorded neurons (9 neurons, 3 animals, one-way analysis of variance Tukey’s multiple comparison two-sided test, ***P = 0.0004, **P = 0.0107, data are presented as mean ± s.e.m.). **m**, Brain schematic showing the location of retrograde transsynaptic vector injection in the vLGN. **n**, Coronal brain sections of transsynaptic EGFP expression by pseudorabies vectors in sensory- and motor-associated areas (*n* = 6 animals). **o**, Summary schematic of vLGN inputs. Zi, zona incerta; S1, primary somatosensory cortex; LM, lateromedial area; V1, primary visual cortex; Cg, cingulate cortex; APT, anterior pretectal area; PAG, periaqueductal gray; LCN, lateral cerebellar nucleus; MVe, medial vestibular nucleus; PPN, pedunculopontine nucleus; Gi, gigantocellular nucleus; Rn, red nucleus; DK, darkschewitsch nucleus. Scale bars, 100 µm. Source data

Vision is constantly challenged by the animal’s own movements^1^. From the image alone, the origins of the sensory perturbations are ambiguous—they could arise from environmental changes (exafference) or the animal’s own movements (reafference) (Fig. 1a). To maintain a coherent perception while moving, animals must distinguish between these sources^2^. This requires sophisticated brain–body coordination coupled with neural mechanisms to actively compensate for visual misalignment during motion. These mechanisms involve multisensory coordination, such as between visual and vestibular systems^3^, and an internal representation of movement commands called efference copy or corollary discharge (CD) (Fig. 1b). CDs filter out reafferent signals, enabling precise sensorimotor transformations essential for inferring external structure^1,4^ and ensuring perceptual continuity^5^. A key example is saccadic suppression in the primate oculomotor system, where CDs signal upcoming saccadic movements to suppress motion-induced blur^5^. However, motion can affect visual processing in myriad ways, making effective estimation and compensation of the reafferent signal complex. This complexity is particularly evident when these corrections are distributed throughout the brain, as observed in mammals, where CDs associated with visual processing have been found in thalamic and cortical regions^1,6^.

The superior colliculus (SC), a conserved midbrain hub across vertebrates^7^, is critical for visual sensorimotor transformations, including in humans^8–10^. The output of the SC has been associated with CDs and has been extensively studied in saccadic suppression^6^. In primates, intermediate SC layers are thought to send motor commands to the medial dorsal thalamus and then to the frontal eye fields in the cortex, a region involved in voluntary saccades^6^. However, the SC has been implicated in a large variety of visuomotor processes, ranging from visual perception to cognition^11^, and some of these processes have been shown to be independent of cortical function^12,13^. Thus, to ensure proper visual perception and visuomotor control via the SC, the SC may not only provide the motor signals required to generate the CD, but would also require processes that allow correction of a range of self-motion-induced visual distortions.

CD signals can emerge at various points along the motor pathway and influence any stage of sensory processing^1^. They serve functions such as compensation, attenuation or suppression^14^, with the latter two often mediated by inhibitory inputs. In the SC, distinct layers are targeted by long-range inhibitory projection from different brain areas^15–18^. Among these, the ventral lateral geniculate nucleus (vLGN), a retino-receptive thalamic region^19^, is well-suited for CD operations. Most neurons are GABAergic^15,16^, connect to major visual brain regions^20^ and participate in diverse tasks, including visual threat response^15,16^, nocifensive behavior^21^, chromatic discrimination^22^, optokinetic reflex^23^ and visuomotor control^20,24–28^. Here, we show in mice that the vLGN acts as a hub-and-spoke network, coordinating brain-wide visuomotor processing. It receives inputs from sensory and motor-related regions and modulates visual responses in the superficial SC (sSC). Furthermore, the vLGN transmits CD signals from behaviors such as locomotion, saccades and pupil dilation to the sSC via strong inhibitory projections, countering temporal and spatial blurring caused by movement. Accordingly, disruption of this fundamental component of proper visual perception impairs tasks requiring vision–action integration. Beyond the sSC, we show that the vLGN projects to several motor control-related areas, forming a distributed spoke-like system that coordinates sensory processing with motor control. As a result, optogenetic activation of the vLGN produces stereotyped corrective movements, whereas targeted suppression disrupts the precision of these behaviors. Taken together, our data show that the vLGN plays a critical role in coordinating visuomotor transformations during action, resembling a distributed feedback control system.

## Results

### The vLGN—an integration hub for sensory and motor areas

The vLGN is a prethalamic nucleus composed of mainly inhibitory neurons^15,16^, forming a tight complex with the intergeniculate leaflet (IGL). Recent work has shown that the vLGN projects across collicular layers (Fig. 1c,d), modulating collicular processing by relaying information of threat levels or feature-related visual signals^16,29^. However, the relative contribution of the vLGN and IGL, in particular to the visual recipient layers, remained contested and the specificity of these projections unknown. To determine the relative contributions of vLGN and IGL^16^ to sSC cell types, we used highly neurotropic N2c rabies virus vectors^30^. We retrogradely labeled the presynaptic neurons of predominantly glutamatergic sSC neurons using the Ntsr1-GN209 (wide-field), Grp (narrow-field) and Rorb-Cre (stellate and other neurons) lines (Fig. 1e,f)^31^. In all cases, we observed predominantly vLGN labeling, with few or no labeled cells in the IGL, and no labeled neurons in the dorsal lateral geniculate nucleus (dLGN) (Fig. 1f). To confirm that the vLGN exerts direct inhibitory control of sSC, we optogenetically stimulated Gad2^+^ vLGN terminals expressing channelrhodopsin-2 (ChR2) in combination with in vitro whole-cell recordings of sSC neurons (Fig. 1g–l). First, we verified that we sampled across a range of cell types, by characterizing the physiological properties of each cell in response to current injections^31^ (Fig. 1h). In neurons that fired spontaneously, sustained optogenetic activation of vLGN terminals led to a near abolishment of spikes (Fig. 1i,j). In voltage-clamp, short optogenetic stimulation (5 ms) reliably led to large, GABA_A_-dependent, inhibitory postsynaptic currents (124 ± 21 pA) (Fig. 1k,l). Overall, these data show that the vLGN is well-positioned to exert a strong modulatory influence on the first visual relay, and likely also across the wide-spread sensory and motor projecting areas^32^.

**Extended Data Fig. 1:**
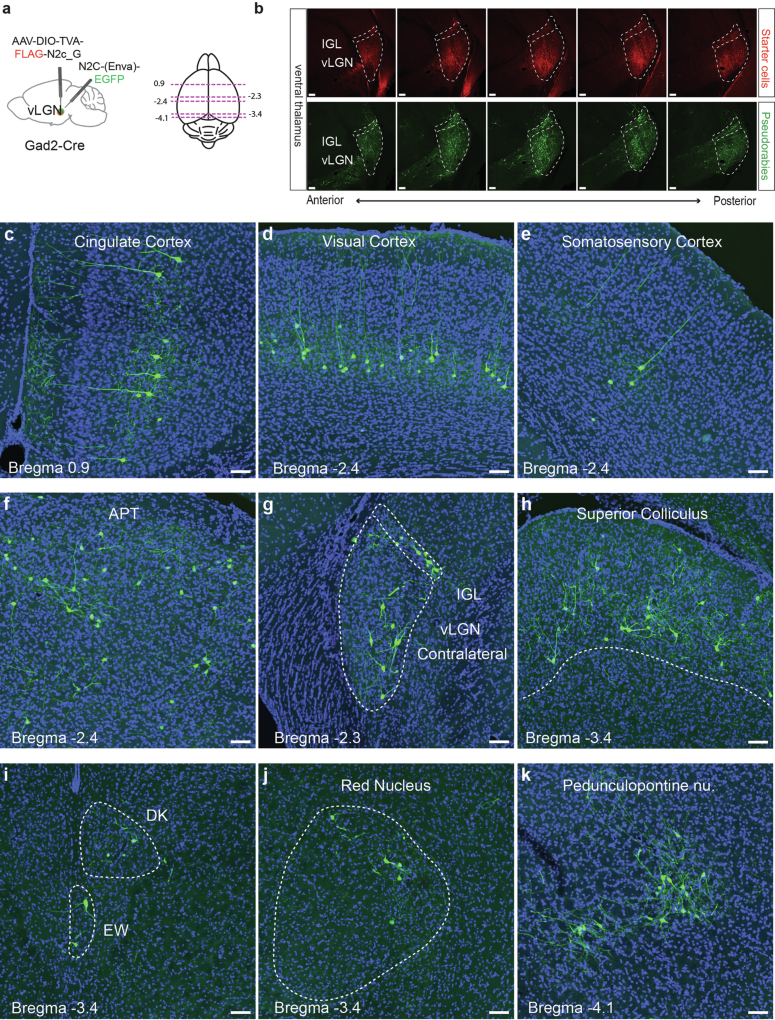
Brain-wide projections to the vLGN reveal an integrative hub, with strong inputs from motor related regions. **a**. Left: Brain schematic showing the location of retrograde transsynaptic vector injections in the vLGN. Right: schematic of a horizontal brain section showing the rostro-caudal locations (magenta dashed line) of the images shown in (**c-k**). **b**. Coronal vLGN series showing the extent of the infection. Top: starter vector expression. Bottom: pseudorabies vector expression, scale bar: 100 µm. (**c-k**) Coronal brain sections of pseudorabies transsynaptic vector expression in sensory- and motor-associated areas, scale bar: 100 µm (n = 3 animals).

Next, using N2c rabies vectors, we mapped the areas providing direct input to Gad2^+^ vLGN neurons (Fig. 1m). In addition to previously characterized inputs from the retina^33^, we observed direct projections from a wide-spread network of subcortical and cortical areas. We found inputs from cortical areas such as visual and cingulate cortical areas, involved in emotional and cognitive control^34^, as well as fear and stress responses^35^ (Fig. 1n,o and Extended Data Fig. 1). We also observed inputs from several pre-motor and motor nuclei, such as red nucleus, pedunculopontine nucleus, gigantocellular reticular nucleus and lateral cerebellar nucleus, which are known to be required for motor coordination (Fig. 1n,o and Extended Data Fig. 1). Interestingly, some of these areas project indirectly or directly^36,37^ to the spinal cord, indicating that copies of motor commands are transmitted directly to the vLGN.

These results demonstrate that the vLGN is situated in the center of an extended network linking motor and sensory areas with inhibitory connections, leading to the hypothesis that it provides CD-type control of visual centers during behavior. Notably, its projection targets prominently include the ipsilateral sSC as a sensory example.

### The vLGN shapes visual responses in the early visual system

**Fig. 2:**
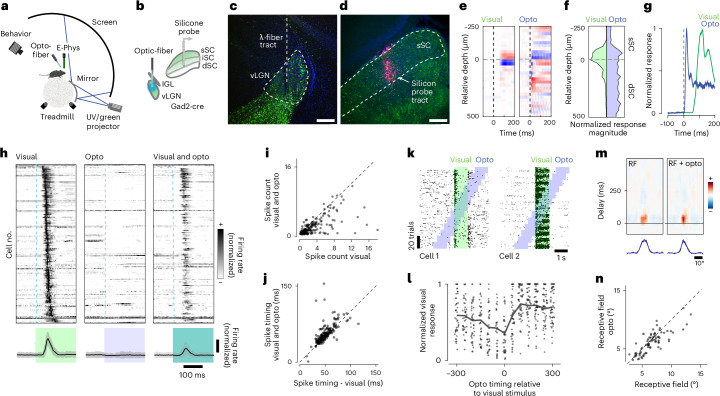
The vLGN modulates the rate, not the timing, in vivo*.* **a**, Schematic of the electrophysiology setup. **b**, Schematic of ChR2 virus infection in Gad2^+^ neurons in the vLGN and placement of the recording electrode. **c**, Confocal image of the vLGN and the optical fiber track for optogenetics. **d**, Confocal image of the SC with the DiI-labeled track of the recording electrode (*n* = 5 animals). **e**, CSD analysis for an example recording for visual flash stimuli (left) and optogenetic stimulation (right). Black vertical dashed line indicates stimulus onset. **f**, Average contours of normalized CSD over depth (26 recordings, 5 animals). **g**, Temporal profile of CSD activation for the recording in **e**. **h**, Sorted and normalized firing responses of sSC units to visual, optogenetic and combined stimulation (top) with their respective population mean responses (bottom shows mean (black) ± s.d. (shaded gray); *n* = 301, 26 recordings, 5 animals). **i**, Quantification of optogenetic activity suppression, spike count within 0.2 s after the flash onset (*n* = 301 units, 26 recordings, 5 animals; two-sided Wilcoxon signed rank test, *P* = 10 × 10^−34^, mean ± s.d. of spike count difference −1.12 ± 2.15). **j**, Analysis of the optogenetic influence on visually evoked spike timing (*n* = 301 units, 26 recordings, 5 animals; two-sided Wilcoxon signed rank test, *P* = 10 × 10^−10^, mean ± s.d. of spike timing difference 3.9 ± 11.9 ms). **k**, Spike raster plots of visually and optogenetically responsive sample units of the sSC. Visual stimulus epochs (green) are interleaved with optogenetic stimulation (blue). **l**, Quantification of the duration of inhibition kinetics, *n* = 81 units, 7 recordings, 4 animals. **m**, Horizontal RF of an example neuron during random vertical bar stimulus (left), with optogenetic stimulation (right) and their horizontal profiles (bottom, data (black) and Gaussian fit (blue)). **n**, Quantification of the difference of sizes of the centers of the RFs with and without optogenetic stimulation. (*n* = 79 cells, 10 recordings, 3 animals; two-sided Wilcoxon signed rank test, *P* = 0.0003, mean ± s.d. of size difference of the center of RFs −0.53 ± 1.23°). Scale bars, 100 µm. dSC, deep SC; mSC, intermediate SC; UV, ultraviolet. Source data

**Extended Data Fig. 2:**
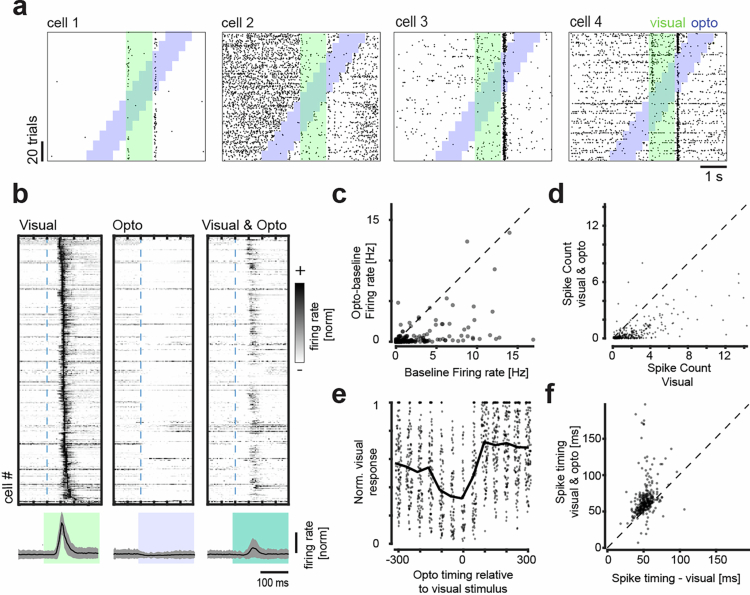
vLGN suppression of intermediate SC *in vivo.* **a**. Spike raster plots of visually and optogenetically responsive sample neurons of the intermediate SC. Visual stimulus epochs (green) are interleaved with optogenetic stimulation (blue). **b**. Sorted and normalized firing responses of intermediate SC neurons to visual, optogenetic, and combined stimulation (top) with their respective mean responses (bottom, mean ± SD). **c-d**. Quantification of optogenetic baseline (c, mean ± SD of firing rate 0.87 ± 2.13) and visually evoked suppression (d, spike count within 0.2 s after the flash onset, mean ± SD of spike count 1.40 ± 1.78) (Wilcoxon signed rank test, p = 10e-33, p = 10e-60). **e**. Quantification of the duration of inhibition kinetics (80 units, 7 recordings, 4 animals). **f**. Analysis of the optogenetic influence on visually evoked spike timing (mean ± SD of spike timing -11.3 ± 22.0 ms, Wilcoxon signed rank test, p = 10e-35). 390 units, 26 recordings, 5 animals if not stated otherwise. Source data

To explore the modulatory influence of vLGN on visual processing, we simultaneously recorded visually evoked responses across different layers of the SC, using silicon probes in head-fixed, awake, behaving mice, and optogenetically activated the vLGN (Fig. 2a,b). For this purpose, we used the same viral delivery approach and Gad2-cre mice as for in vitro physiology (Fig. 1g) and subsequently implanted an optical λ-fiber for optogenetic stimulation (Fig. 2c,d). First, we determined the recording depth using visually evoked responses and current source density (CSD) analysis (Fig. 2e), which was aligned with the histological reconstruction of the probe position (Fig. 2d). When visual stimuli were replaced by optogenetic pulses in the vLGN, we observed an inversion of the current source, reflecting the inhibitory nature of the thalamic projection (Fig. 2e). The response depth of the visual and optogenetic responses overlapped, but the optogenetic CSDs reached further into the intermediate layers (Fig. 2f) as expected from the anatomical projection. Visual responses peaked at ~60 ms after the visual stimulus onset likely due to the speed of the phototransduction, whereas optogenetic responses had a peak-latency of a few milliseconds (Fig. 2g). To quantify how these pronounced inhibitory dynamics influence visual responses in the sSC, we combined small visual flashes (10° of visual angle, duration 200 ms or 1 s) centered in the receptive fields (RFs) of the recorded sSC units with interspersed and randomized vLGN optogenetic stimulation (Fig. 2h). Next, we determined the units that were visually and optogenetically responsive, independent of variations in retinotopic position, using parameter-free stimulus-evoked responsiveness tests to directly examine interactions. We found that 66% (376 of 571) of all recorded units were visually responsive, of which 67% (253 of 376) were responsive to optogenetic stimulation. On average, this population showed a mild modulation of baseline firing (Fig. 2h), but a strong and effective suppression of visually evoked responses (Fig. 2h,i), reducing the maximum firing rate by ~60% (Fig. 2h,i). Since the relative timing of the first spike has been shown to be an effective retinal code^38^, we next asked whether feedforward suppression would affect spike timing. We tested spike timing precision on the subset of units that did not have complete suppression and compared the latency of the first spike with a visual stimulus between control and optogenetically stimulated trials. On average, optogenetic stimulation had a small effect, delaying the first spike by 3.9 ms (Fig. 2j), indicating that vLGN inhibition in the SC mostly affects the rate but not the timing of the first spike. To determine the kinetics of inhibition, we sorted the trials by the relative onset time of the optogenetic stimulation (Fig. 2k). vLGN suppression was largely transient, was strongest when visual and optogenetic stimuli overlapped and lasted for approximately 100 ms after the offset of the optogenetic stimulus (Fig. 2l). Consistent with the extension of vLGN projection to lower SC layers^15,16^, we observed similar suppression in intermediate SC layers (Extended Data Fig. 2). Finally, we tested whether vLGN activation would modulate sensory properties such as the spatiotemporal RFs of sSC neurons. We mapped the one-dimensional RF using vertical bars appearing at random horizontal locations interleaved with optogenetic stimulation. As shown previously^29^, we observed a sharpening of the spatial RF (Fig. 2m,n).

Collectively, these data show that the vLGN acts as a feedforward inhibitory hub capable of strongly reducing visual responses across the sSC, while leaving onset of timing largely unaffected, thus effectively sharpening visual responses in both spatial and temporal domains.

### vLGN is a central hub for CDs

**Fig. 3:**
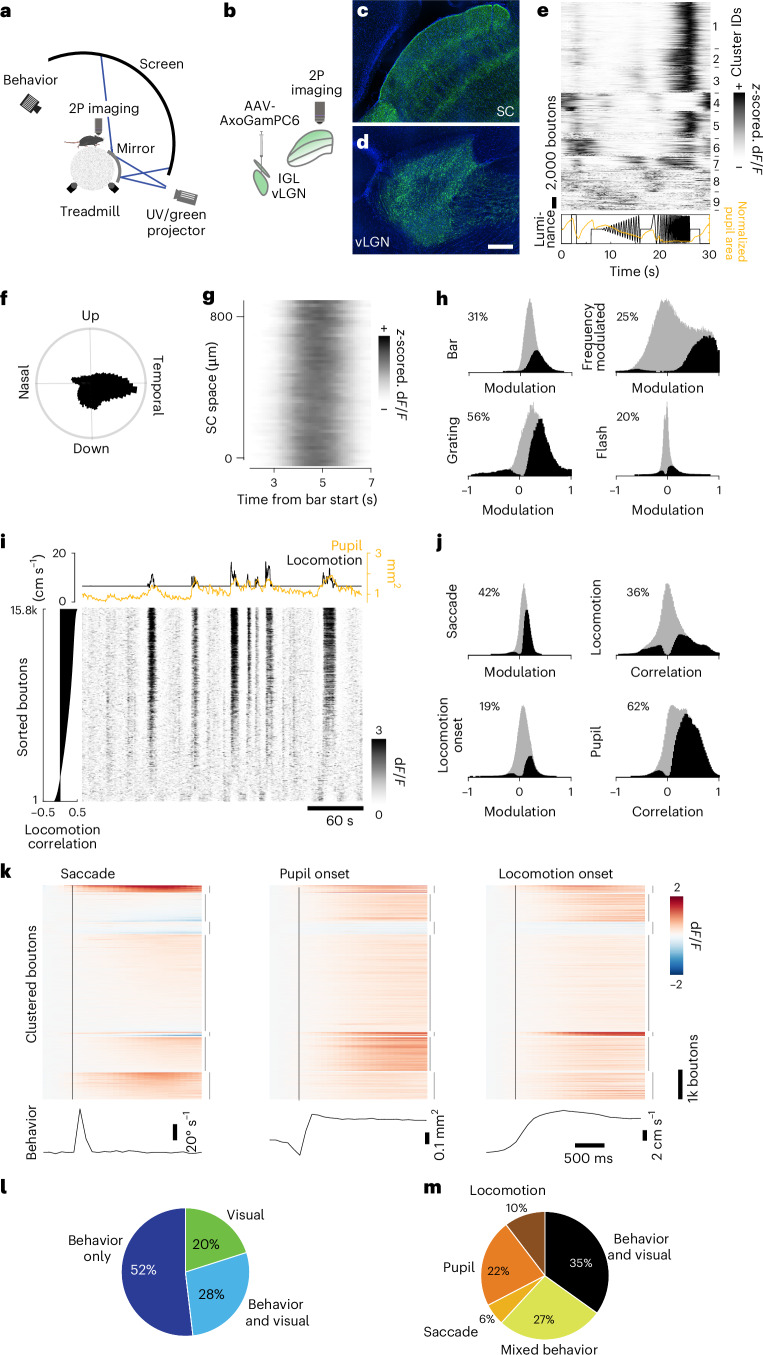
vLGN to SC projections are strongly modulated by behavior. **a**,**b**, Multiphoton imaging setup (**a**) and viral expression schematic (**b**). **c**,**d**, Confocal images of axonal terminals in the sSC (**c**) and the infection area in the vLGN (**d**), sagittal view. Scale bar, 100 µm; *n* = 6 animals. **e**, Cluster-sorted, averaged and *z*-scored responses (top) to full-field luminance chirps (bottom, black line) of vLGN boutons in the sSC and average normalized pupil area (bottom, orange line). **f**, Polar histogram of direction-selective (*P* < 0.01, shuffle test, *n* = 31,620 (26%)) boutons’ preferred grating direction. **g**, Example recording with *z*-scored and spatially binned (20 µm) vLGN bouton (SNR > 0.35, *n* = 38,012, 29 recordings, 6 animals) responses across SC space to a moving bar, moving in nasal-temporal direction. **h**, Histograms of modulation indices of vLGN bouton population (gray) and significantly modulated boutons (black; two-sided Wilcoxon signed rank test, *P* < 0.01) to visual stimuli. **i**, Raster plot of example recording of vLGN boutons sorted by correlation with locomotion (left) and respective locomotion speed (black) and pupil area (orange, top). **j**, Histograms of modulation indices (saccades, locomotion onset) or correlation coefficients (locomotion speed, pupil area) of vLGN/IGL bouton population (gray) and significantly modulated boutons (black and percentages) to behavioral parameters. *P* < 0.01, two-sided Wilcoxon signed rank test, shuffle test for correlations (Methods). **k**, Average modulation of vLGN boutons (*n* = 131,871, 17 recordings, 5 animals) aligned to saccades (bottom, average eye movement speed), rapid pupil expansion onset (bottom, average pupil area) and locomotion onset (bottom, average forward locomotion speed). Time of behavioral event indicated by vertical black line. Boutons sorted by *k*-means clustering (interrupted vertical lines on the right, *k* = 7) joint activity profiles. **l**, Pie chart of boutons significantly modulated by any of the visual stimuli tested (**h**) and/or significantly modulated by/correlated to any of the behavioral parameters (see **j**); *P* < 0.01, Bonferroni-corrected for multiple comparisons. **m**, As **i**, but removing purely visually responsive neurons (*n* = 176,282, 38 recordings, 6 animals); *P* < 0.01, Bonferroni-corrected for multiple comparisons. *n* = 220,021, 38 recordings, 6 animals, if not stated otherwise. 2P, two-photon. Source data

**Extended Data Fig. 3:**
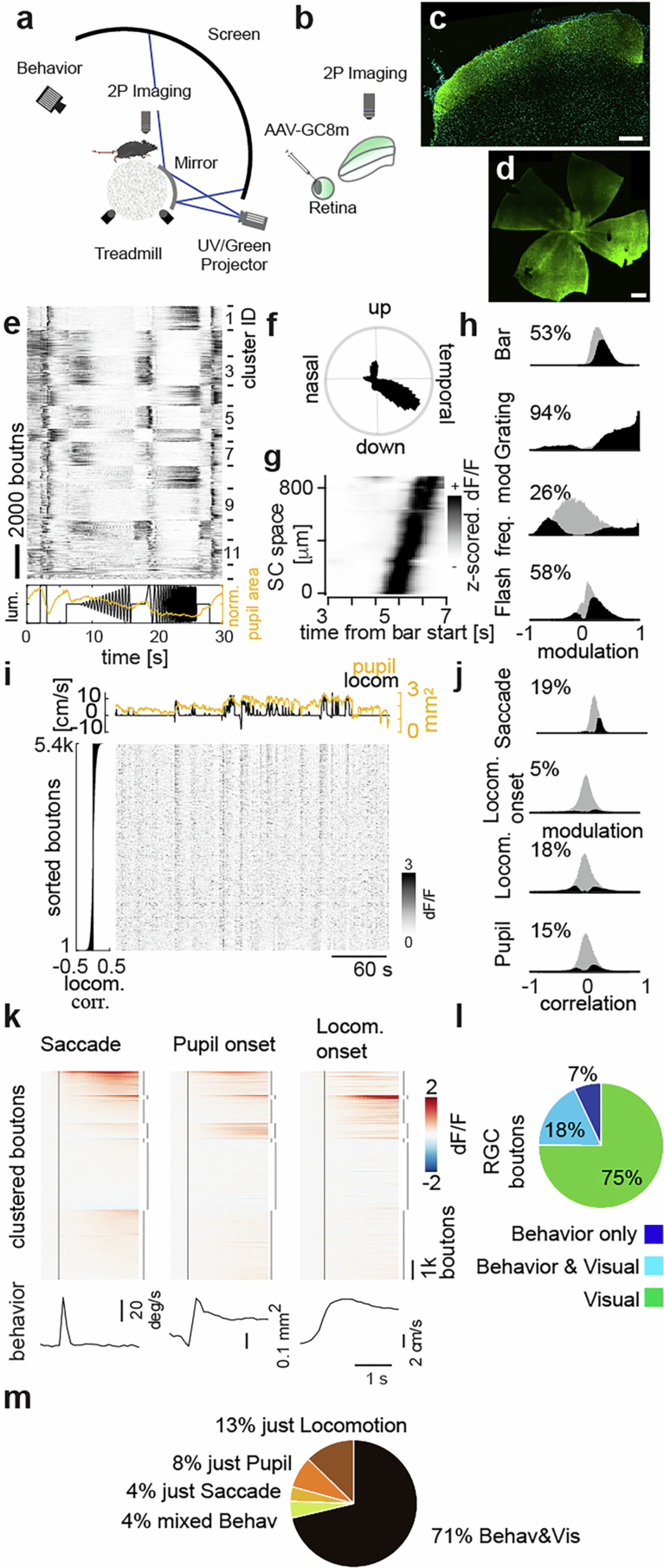
Retinal ganglion cell (RGC) activity in superficial SC is primarily visual. **a**. Schematic of the imaging setup and (**b**) subretinal GCaMP8 infection and imaging approach. **c**. Confocal micrograph of the SC (scale bar: 500 µm) and (**d**) an infected retina (scale bar: 200 µm). **e**. Cluster-sorted, averaged and z-scored responses (top) to full field luminance chirps (bottom, black line) of RGC boutons (SNR > 0.35, n = 15941, 15 recordings, 4 animals) in the sSC and average modulation of normalized pupil area (bottom, orange). **f**. Polar histogram of direction-selective (p < 0.01, shuffle test, n = 40471 (64%)) RGC boutons’ preferred direction in response to full field drifting gratings. **g**. Z-scored and spatially binned (20 µm) bouton responses to moving bar across SC space. **h**. Histograms of modulation indices of RGC bouton population (gray) and significantly modulated boutons (percentages, black) to different visual stimuli. Two-sided Wilcoxon signed rank test, p < 0.01. **i**. Raster plot of example recording of RGC boutons sorted by correlation with locomotion (left) and respective locomotion (black, top) and pupil area (orange, top) traces. **j**. Histograms of modulation indices (saccades, locomotion onset) or correlation coefficients (locomotion speed, pupil area) of RGC bouton population (gray) and significantly modulated boutons (percentages, black) to behavioral parameters. Two-sided Wilcoxon signed rank test, shuffle test for correlations (see methods), p < 0.01. **k**. Average activity modulation of RGC boutons (n = 40389, 19 recordings, 4 animals, only including recordings with at least 10 trials per each condition) aligned to saccades (bottom: average eye movement speed), rapid pupil expansion onset (bottom: average pupil area) and locomotion onset (bottom: average forward locomotion speed). Time of behavioral event indicated by vertical black line. Boutons sorted by k-means clustering (interrupted vertical lines on the right, k = 7) joint activity profiles. **l**. Pie chart of boutons significantly modulated by any of the visual stimuli tested (**h**) and/or significantly modulated by/correlated to any of the behavioral parameters (**j**); p < 0.01, Bonferroni corrected for multiple comparisons. **m**. as (**l**), but removing purely visually responsive neurons (n = 19866, 29 recordings, 5 animals); p < 0.01, Bonferroni corrected for multiple comparisons. n = 78730, 29 recordings, 5 animals, if not stated otherwise. Source data

To investigate the scenarios in which the vLGN modulates sSC visual processing, we analyzed the response properties of vLGN axon terminals in the sSC (Fig. 3a). We introduced the calcium indicator axon-GCaMP6s^39^ into Gad2^+^ cells by infecting the vLGN (Fig. 3b–d; *n* = 4 mice), resulting in homogeneous axonal GCaMP expression throughout the SC (Fig. 3c). Subsequent implantation of a cranial window over the SC allowed observation of activity in axonal terminals from the thalamus using two-photon calcium imaging in awake, behaving mice. In separate experiments, retinal terminals in the SC were recorded to qualitatively compare their response characteristics (Extended Data Fig. 3). We used a variety of visual stimuli to assess bouton response properties, with interleaved periods of uniform light level (gray dome screen) to assess the activity associated with spontaneous animal behavior.

First, we tested bouton responses to a ‘chirp’ stimulus^40^, which involves full-field modulations of light intensity (Fig. 3e). Among all vLGN boutons meeting the inclusion criteria (Methods), some showed robust responses to full-field flashes, as previously reported^41^. However, the majority showed their most pronounced responses to rapid frequency modulations, which can occur during locomotion, such as running underneath nearby vegetation. Retinal boutons, on the other hand, showed a wider range of feature selectivities to the stimulus (Extended Data Fig. 3e). Next, we determined directional selectivity to full-field moving grating stimuli, which in vLGN boutons showed a striking preference for temporal grating motion (Fig. 3f), as would be seen during forward locomotion. To determine the retinotopic organization of the terminals in the sSC, we tested whether a visual bar moving slowly (22.5° s^−1^) across the visual field elicited sequential activations that were anatomically localized. Retinal synapses showed a distinct spatial representation (Extended Data Fig. 3g), whereas vLGN terminals showed global responses when a bar crossed visual space, consistent with electrophysiological data indicating large spatial RFs^41^ (Fig. 3g). Overall, approximately half (48%) of the included vLGN boutons (retinal ganglion cells (RGCs): 93%) responded significantly (*P* < 0.01, Bonferroni-corrected) to at least one of the visual stimuli presented (Fig. 3h).

**Extended Data Fig. 4:**
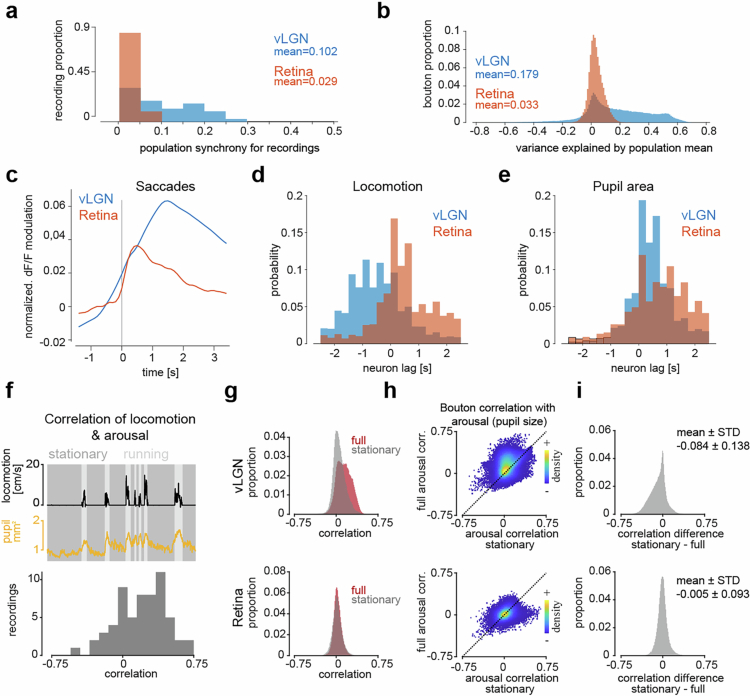
Synchrony, timing, and behavioral dependencies of vLGN and retinal terminals in the superior colliculus. **a**. histograms of bouton activity synchrony of vLGN/IGL (blue, n = 38 recordings) and RGC (red, n = 29) boutons within each recording. **b**. histograms of explained variance by the population mean for vLGN/IGL and RGC boutons. **c**. mean dF/F modulation of vLGN and RGC boutons following spontaneous saccades. Note: while the response kinetics are not directly comparable between the populations due to different indicators (GCaMP8m for RGCs and GCaMP6s for vLGN), the normalization provides relative contribution of saccadic responses. **d**. histograms of cross-correlation peak lags for significantly positively correlated vLGN/IGL (n = 75915, 35%) and RGC (n = 16122, 21%) boutons with locomotion speed. **e**. same as (**d**) but for cross-correlations with pupil area (vLGN/IGL: n = 140794, 64%; RGC: n = 15857, 20%)) **f**. top, example trace of correlated locomotion (black) and pupil size (yellow). Periods of no locomotion (speed < 1 cm s^-1^, “stationary”, dark gray) were selected for comparisons in (g-i). Bottom, histogram of correlation coefficients between pupil size and locomotion over pooled recordings (n = 67 recordings). **g**. Normalized histograms of overall (red) correlation coefficients for vLGN (top) and RGC (bottom) boutons with pupil size as well as correlation to pupil size in stationary periods (gray). **h**. Density-colored scatter plots of bouton full correlation coefficients over stationary correlation coefficients, as in b, for vLGN (top) and RGC boutons (bottom). Two-sided Wilcoxon signed-rank test. Gray screen periods selected for (**a**-**h**). **i**. histogram of differences between stationary and full correlation coefficients, showing a strong reduction for vLGN (top) and a small reduction for RGCs (bottom). (**b-e**), (**g-i**) vLGN: n = 220021 boutons, RGC: n = 78730. Source data

During gray screen periods, activity in retinal boutons was weak and sparse (Extended Data Fig. 3i), whereas calcium signals in vLGN boutons varied strongly (Fig. 3i). Compared with the retinal terminals, vLGN bouton activity had a high degree of synchrony (on average, 18% variance explained by population mean for vLGN, 3% for RGCs; Extended Data Fig. 4a,b), indicating that the vLGN is transmitting a global signal. We found that activity peaks often coincided with locomotor bursts and thus compared vLGN bouton signals with behavioral parameters. Indeed, many vLGN boutons significantly changed their activity at saccade, pupil dilation and locomotion onset (Fig. 3j,k). Confirming previous results^42^, retinal inputs to the sSC were also modulated by behavior during gray periods, with a quarter of retinal boutons significantly affected by at least one of the measured behavioral parameters (Extended Data Figs. 3j–l and 4c). However, vLGN bouton activity was coupled to behavior to a much greater extent and proportion, with 80% of boutons significantly and mostly positively modulated by behavior (Fig. 3l), in stark contrast to retinal axons (Extended Data Fig. 3l). Interestingly, ~38% of behaviorally modulated vLGN boutons were specifically modulated by only one behavioral parameter (Fig. 3m), suggesting that the vLGN is composed of specific cell types carrying distinct information, in line with single-cell sequencing data of the vLGN reporting a large neuronal diversity^43^. Since the dynamics of locomotion and pupil size can be coupled (median correlation across recordings *c* = 0.24, *n* = 67 recordings; Extended Data Fig. 4f), we next tested whether vLGN bouton activity coupling to each behavior is independent. We first tested the relative neuron-behavior cross-correlation timing, determining that correlation to running speed was maximal when vLGN bouton activity was shifted to slightly preceding the behavior (Extended Data Fig. 4d), in contrast to pupil area, where minimally delayed neuronal activity led to maximal correlations (Extended Data Fig. 4e). To further uncouple pupil size and locomotion in our analyses, we compared the difference of correlation of vLGN and retinal activity with pupil size overall and during stationary epochs only. We found that in stationary periods pupil size correlations were significantly lower for both bouton populations, but to a far larger extent in vLGN boutons (mean difference 0.084 ± 0.138 s.d.) than RGC boutons (mean difference 0.005 ± 0.093 s.d.), indicating that locomotion and pupil dynamics exert an independent influence on vLGN boutons (Extended Data Fig. 4g–i). In conclusion, the vLGN to sSC projections are activated by cross-modal signals, that is, visual and behavioral. This activity is well-positioned to provide a potent inhibition of visual signals when changes in the retinal image are expected to occur due to self-motion, for example, to counteract luminance changes during pupil dilation or motion blur during locomotion or saccades.

### The vLGN coordinates visual and motor signals

**Fig. 4:**
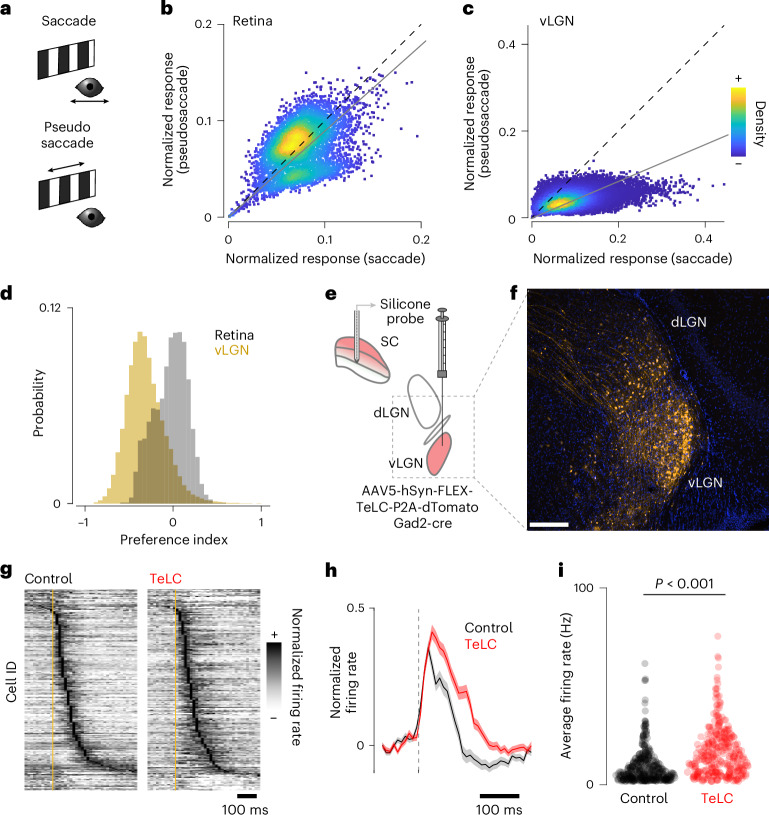
The vLGN suppresses behaviorally induced sensory blur. **a**, Spontaneous saccades on a stationary grating or checkerboard (‘saccades’, top, black) and saccade-like shifts of the displayed texture during stationary eye periods (‘pseudosaccades’, bottom, green) induce comparable image shifts. **b**, Density-colored scatter plot of normalized responses of individual retinal boutons comparing pseudosaccades and saccades, showing stronger responses to pseudosaccades (linear fit: gray line, slope = 0.89, *P* = 5.5 × 10^−14^, paired, two-tailed Wilcoxon signed rank test, *n* = 5,174). **c**, Same as **b** but for vLGN boutons with a strong preference for saccadic motion (linear fit: gray line, slope = 0.42, *P* < 10 × 10^−130^, paired, two-tailed Wilcoxon signed rank test, *n* = 51,996). **d**, Histograms of the bouton-wise pseudosaccade preference index for RGC (gray) and vLGN (yellow) bouton responses as in **b** and **c**, with vLGN bouton preference shifted to the negative compared with RGC (*P* < 10 × 10^−130^, two-tailed Wilcoxon rank sum test, vLGN: mean −0.310 ± 0.214 s.d., RGC: mean −0.030 ± 0.187 s.d.). **e**, Schematic of virally mediated TeLC expression in vLGN and SC extracellular recording. **f**, Confocal micrograph of TeLC-tdTomato expression (orange) in thalamus of Gad2-cre mice. Scale bar, 100 µm; *n* = 4 animals. **g**, Normalized peristimulus time histogram (PSTH) of mean saccade-triggered responses in control (left) and TeLC (right) mice (control *n* = 370 units, 8 recordings, 5 animals; TeLC *n* = 295 units, 6 recordings, 4 animals). **h**, Normalized population-averaged saccade responses and s.e.m. (shading). **i**, Average firing rate for the first 200 ms after saccade onset for all units in **g** (*P* = 10 × 10^−18^, KS (Kolmogorov-Smirnov) test). Source data

**Extended Data Fig. 5:**
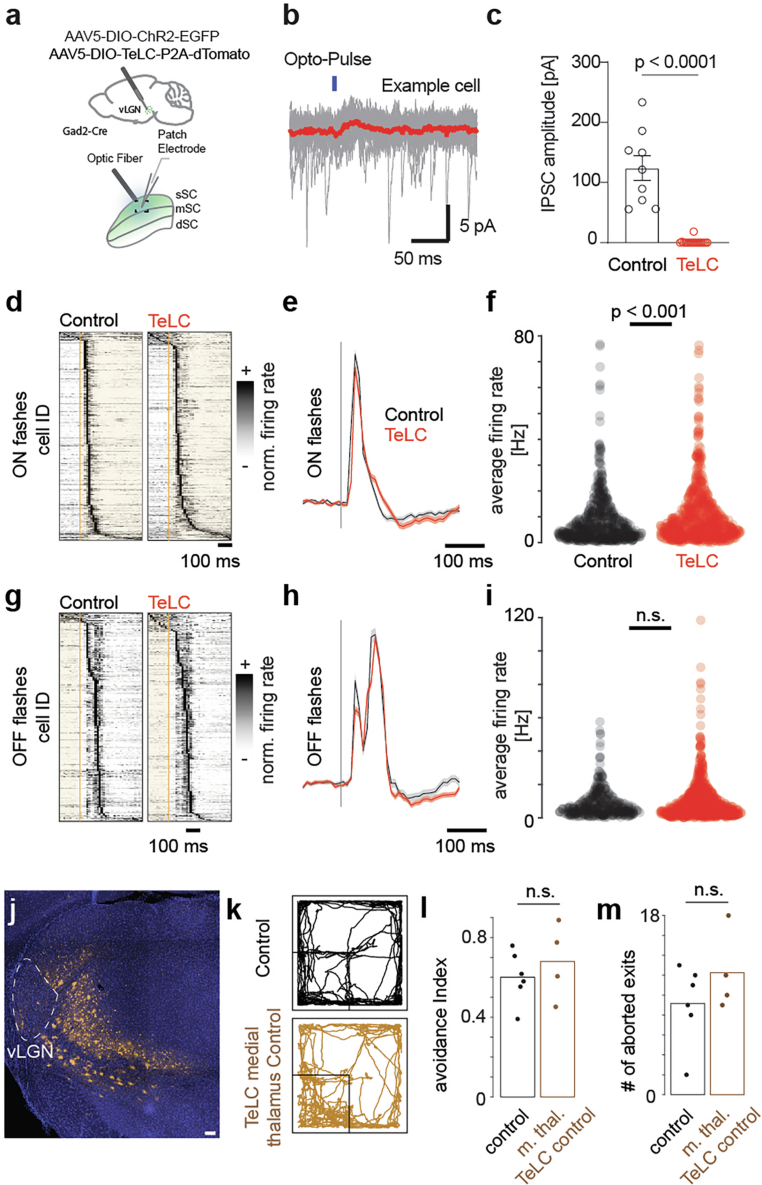
TeLC control experiments. **a-c**. TeLC effectively blocks synaptic transmission of the vLGN. **a**. Schematic of a combined viral infection of TeLC and ChR2 in the vLGN/IGL complex and subsequent patch clamp recordings in the sSC. **b**. Example trace of optogenetically activated IPSC in sSC neurons with TeLC block (average in red). **c**. Quantification of ChR2 evoked IPSC current amplitude with and without TeLC block (Control: 9 cells, 3 animals, TeLC: 17 cells, 2 animals; Mann-Whitney U two-sided test, p = 0.000005, data presented as mean values +/- SEM). **d-i** vLGN block has a minor effect on visual responses to flashes in the SC (ON: control: 659 units, 7 recordings, 5 animals; tnt: 759 units, 10 recordings, 8 animals; OFF: control: 421 units, 7 recordings, 5 animals; tnt: 866 units, 10 recordings, 8 animals). **d**. Normalized PSTH of responses to ON flashes in control (left) and TeLC (right) mice. **e**. Normalized population-averaged ON flash responses and SEM (shading). **f**. Average firing rate for the first 200 ms after ON flash onset (KS test, p = 10e-5; mean ± SD: control = 8.26 ± 9.64, tnt = 10.07 ± 10.84). **g-i**. as (**d-e**) but for OFF flashes (**i**) (KS test, p = 0.2; mean ± SD: control = 10.19 ± 10.28, tnt = 11.74 ± 14.3). **j-m**. TeLC expression medial to the vLGN (including ZI), does not affect cliff avoidance (6 control and 4 TeLC medial thalamus control mice). **j**. Location of the viral expression for TeLC-tdTomato expression (orange) in Gad2-cre mice. Scale bar: 100 µm, (n = 4 animals) **k**. Examples of running trajectories during the first 10 min for control and TeLC medial thalamus control mice. **l-m**. Quantification of behavior per animal (right-tailed Wilcoxon rank sum test, p-values 0.82 and 0.83 for l and m). Source data

Our anatomical and physiological data (Figs. 1–3) suggest that the vLGN acts as a feedback controller, anticipating the effects of movement on the animal’s visual input. Thus, this pathway should be required to minimize movement-related visual blur, for example, being involved in saccadic suppression, and for maintaining perceptual stability during behavior. We tested this hypothesis by comparing vLGN responses to saccades while displaying a stationary structured background with visually evoked responses to ‘pseudosaccades’ (movements of the displayed pattern that would mimic the visual input perceived during saccades; Fig. 4a)^44^. As expected, retinal boutons in the SC responded similarly, irrespective of whether the eye or visual stimulus moved (Fig. 4b). In contrast, vLGN boutons were mainly sensitive to real saccades (Fig. 4c), showing a clear preference for voluntary eye movement saccades over pseudosaccades (Fig. 4d). Next, to directly test whether the vLGN/sSC network functions as a feedback control loop, we chronically blocked bilateral Gad2^+^ vLGN output by expressing tetanus toxin light chain (TeLC)^45^ (Fig. 4e,f) and recorded sSC electrophysiologically as previously (Figs. 1 and 2). First, we confirmed in vitro that TeLC blocks synaptic release (Extended Data Fig. 5a–c). We then verified in vivo that behaviorally independent visual responses in SC were largely unaltered (Extended Data Fig. 5d–f). Next, we tested whether the vLGN is required to reduce the motion blur relayed by the retina during saccades while viewing a high-contrast screen. Indeed, in animals in which the vLGN output was blocked, sSC responses to saccades were on average 100 ms longer (Extended Data Fig. 4g,h) and showed increased average firing rates in the first 200 ms (Fig. 4i). Thus, such action-induced feedback can be thought of as a neural mechanism that reduces the effective visual exposure time during actions.

### vLGN feedback is required for visual perception in action

**Fig. 5:**
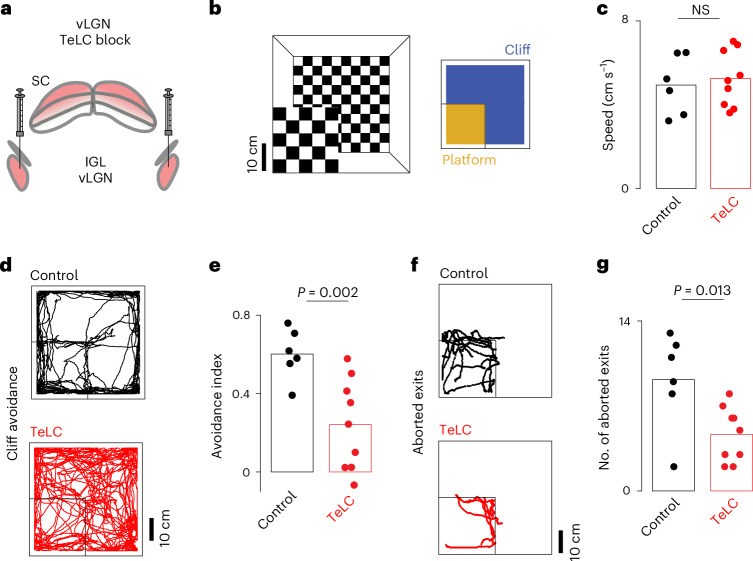
The vLGN modulates visual percepts during voluntary movements. **a**, Schematic of the bilateral TeLC infection of vLGN. **b**, Schematic of the visual cliff setup (left) and areas used for subsequent quantification (right). **c**, Average running speed of control and TeLC mice in the arena (Wilcoxon rank sum test). **d**, Examples of running trajectories during the first 10 min for control and TeLC mice. **e**, Cliff avoidance quantification (Methods) of **d** per animal (right-tailed Wilcoxon rank sum test). **f**, As **d**, but only showing trajectories for aborted exits (2 s before and after a detected crossing with the subsequent reversal of the motion velocity; Methods). **g**, The corresponding quantification (right-tailed Wilcoxon rank sum test). *n* = 6 control and *n* = 9 TeLC animals; each dot represents an animal. NS, not significant. Source data

Our previous results suggest that the vLGN is required for the brain’s ability to interpret visual signals during motion. Recently, it has been shown that mice can use monocular and binocular cues to estimate depth^46^ and that monocular perception requires motion for proper depth estimation^47^. We therefore decided to test depth perception using a classic visual cliff paradigm (Fig. 5b), which relies on monocular visual activity from the lower visual field, where there is minimal binocular overlap^46,47^. For these experiments, to minimize tactile sensory input, we clipped the whiskers of TeLC-mediated (Fig. 5a) bilaterally vLGN-blocked and control mice, placed the animals individually on the platform and recorded their behavior. Control and TeLC mice showed no difference in their average running speed while exploring the arena (Fig. 5c), indicating that chronically blocking vLGN did not grossly change locomotor and general exploratory behavior. Control mice showed a strong preference for the platform, but also roamed around the edges of the arena, possibly touching the walls with their bodies (Fig. 5d). We therefore restricted our analyses to the central part of the arena. vLGN-blocked mice showed a strong reduction in cliff avoidance (Fig. 5d,e). In line with these findings, we observed that control animals more frequently aborted movements out of the platform, compared with vLGN-blocked mice (Fig. 5d,g), suggesting that vLGN-blocked animals have difficulty judging depth. This impairment is linked to an increase in visual blur during motion caused by various behaviors when the vLGN is blocked (Fig. 4), affecting motion parallax computation. Finally, TeLC expression in adjacent medial thalamic areas, including the zona incerta, did not affect cliff avoidance behavior, indicating that the vLGN is specifically required (Extended Data Fig. 5g–j). Taken together, these results show that visuomotor processing and perception during self-generated motion are impaired in the absence of proper vLGN function.

### vLGN distributed projections drive corrective actions

**Fig. 6:**
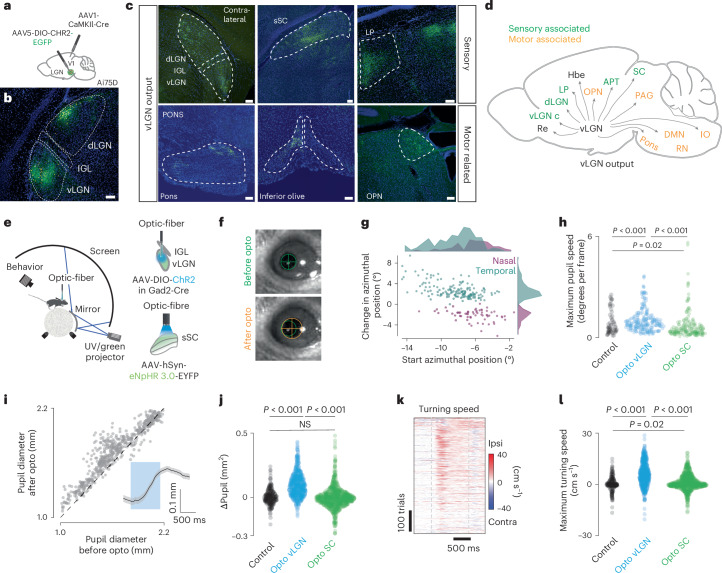
Brain-wide vLGN projections fine-tune behavior. **a**, Schematic showing the location of anterograde transsynaptic vector injection. Primary visual cortex (V1) and vLGN (green dot) in Ai75D mice, which express nuclear-localized tdTomato following Cre recombinase exposure. **b**, Brain section showing ChR2-EGFP expression after Cre-dependent vector injection in the vLGN (*n* = 3 animals). **c**, Expression of ChR2 in vLGN terminals in sensory and motor-associated areas. **d**, Schematic summary of vLGN outputs. **e**, Left, schematic of the setup with head-restrained mice while running on a spherical treadmill. Right, schematic of vLGN (top) and sSC (bottom) optogenetic stimulation. **f**, Example of an optogenetically induced saccade. Top, before optogenetic stimulation; bottom, after. Green and orange circles demarcate the size and position of the pupil, before and after stimulation. **g**, Magnitude and direction of optogenetically induced saccades (only saccades that exceeded a speed threshold were plotted (Methods), 211 repetitions, 3 animals, 9 recordings, each dot represents one saccade). **h**, Quantification of optogenetically induced pupil velocity (two-sample KS test, repetitions, recordings, animals for control: 62, 3, 2; opto vLGN: 211, 9, 3; opto SC: 185, 3, 3; *P* = 10 × 10^−5^; 10 × 10^−16^; 0.02). **i**, Optogenetically induced pupillary dilation. Inset, average kinetics (*P* = 10 × 10^−75^, Wilcoxon signed rank test, 544 repetitions, 11 recordings, 4 animals). **j**, Quantification of optogenetically induced pupil change (two-sample KS test, repetitions, recordings, animals for control: 229, 3, 2; vLGN: 546, 11, 4; SC: 779, 3, 3; *P* = 10 × 10^−39^; 10 × 10^−80^; 0.053). **k**, Optogenetically induced turning speed. Gray dashed lines represent the start and end of the stimulation (546 trials, 11 recordings, 4 animals). **l**, Quantification of optogenetically induced turning speed for control, vLGN and SC (two-sample KS test, repetitions, recordings, animals for control: 229, 3, 2; vLGN: 546, 11, 4; SC: 780, 3, 3; *P* = 10 × 10^−35^; 10 × 10^−52^; 0.019,). Scale bars, 100 µm. Re, nucleus reuniens; vLGN c, vLGN contralateral; LP, lateral posterior nucleus; Hbe, habenula; OPN, olivary pretectal nucleus; IO, inferior olive; DMN, deep mesencephalic nucleus. Source data

**Extended Data Fig. 6:**
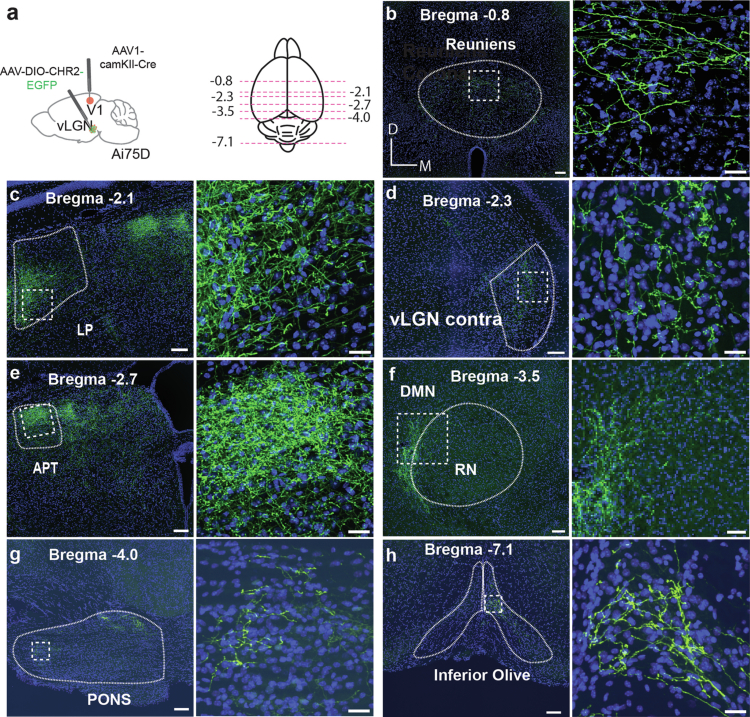
Brain-wide vLGN projections using the AAV1-Cre intersectional approach. **a**. Left: Brain schematic showing the location of anterograde transsynaptic vector injection. Primary visual cortex (V1, red dot) and ventral lateral geniculate nucleus (vLGN, green dot) in Ai75D mice, which express nuclear-localized tdTomato following Cre recombinase exposure. Right: Schematic of coronal confocal images of vLGN terminals. **b-h**. left: low magnification, right: close ups. (**b**) Nucleus reuniens, (**c**) Lateral posterior nucleus (LP), (**d**) Contralateral vLGN, (**e**) Area pretectalis (APT), (**f**) Deep midbrain nucleus (DMN) and red nucleus (RN), (**g**) Pons; right, high magnification of box in (**e**), (**h**) Left, inferior olive (IO); right, high magnification of box in (**f**). Low magnification images scale bars: 100 µm; close up images scale bar: 50 µm, (n = 3 animals).

**Extended Data Fig. 7:**
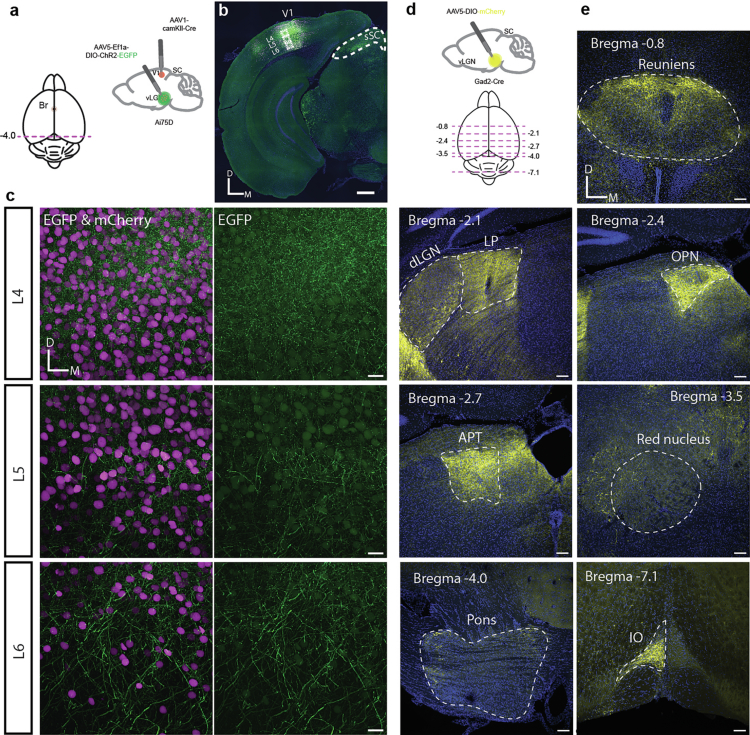
AAV-Cre control experiments and supporting anatomical evidence of vLGN projections using Gad2-Cre expression. **a-c**. Anterograde AAV1-Cre mediated intersectional approach, initiated in the visual cortex, is specific for the visual thalamus. **a**. Top right, a schematic of a sagittal brain showing the location of anterograde transsynaptic vector injection in the primary visual cortex (V1, red dot) and Cre-dependent vector injection in the ventral lateral geniculate nucleus (vLGN, green dot). Bottom left, a schematic of a horizontal brain section showing the rostro-caudal locations (magenta dashed line) of the images shown in (**b,c**). **b**. Confocal image of the injection site in primary visual cortex (V1), scale bar: 500 µm (n = 3 animals). **c**. High-magnification confocal images of V1 across cortical layers (L4, top; L5, middle; L6, bottom). All scale bars: 20 µm. **d-e**. Brain-wide vLGN projections using targeted infections in Gad2-Cre animals. **d**. Top right, schematic of a sagittal brain showing the location of anterograde vector injection into the vLGN; bottom left, schematic of a horizontal brain section showing the rostro-caudal locations (magenta dashed line) of the vLGN efferents and the injection site. **e**. Coronal confocal images of vLGN terminals of nucleus reuniens, Lateral posterior nucleus (LP), Olivary pretectal nucleus (OPN), Area pretectalis (APT), Pons and Inferior olive (IO). Injection site in vLGN and projections to the superior colliculus are shown in Fig. 6. Scale bars: 100 µm (n = 3 animals).

Several classical studies indicate a wide-spread connectivity architecture, yet have not distinguished vLGN projections from those in the IGL^32^ and zona incerta. To determine the brain-wide projections that arise specifically from the vLGN, we performed anterograde labeling experiments (Fig. 6a). We isolated vLGN neurons by transsynaptically labeling visual cortex target neurons, which in the thalamus includes the vLGN and the dLGN, but excludes IGL (Fig. 6b). We did not observe any retrogradely labeled L5 pyramidal cells in the visual cortex (Extended Data Fig. 7a,c). Corroborating our previous findings (Fig. 1), vLGN projections to the sSC were directed to the lower layers of the sSC (Fig. 6c), where most somata of retinorecipient neurons are located^31^. We also observed projections to the intermediate layers of the SC^15,16^, but most prominently a wide projection pattern to a range of subcortical sensory and motor-related areas. Targeted sensory areas are located in the thalamus (lateral posterior nucleus, nucleus reuniens, contralateral dLGN and vLGN, lateral habenula) and midbrain (posterior pretectal nucleus, anterior pretectal nucleus, olivary pretectal nucleus, periaqueductal gray) (Fig. 6c and Extended Data Fig. 6). Projections to motor-related areas include midbrain and hindbrain structures (Fig. 6c and Extended Data Fig. 6); for example, the pretectal olivary nucleus, an area known to directly activate the Edinger–Westphal nucleus, the strongest regulator of pupillary constriction^48^. The vLGN also projects to the pons and inferior olive, involved in visuomotor coordination^49^, the deep mesencephalic nucleus, known as an output center of basal ganglia^50^, and to the red nucleus, an area suggested to be involved in fine motor coordination^51^. These projection patterns can be recapitulated by targeting Gad2^+^ cells in the vLGN/IGL (Extended Data Fig. 7d,e)^32^, indicating that GABAergic neurons in vLGN are the major contributors to these projections.

**Extended Data Fig. 8:**
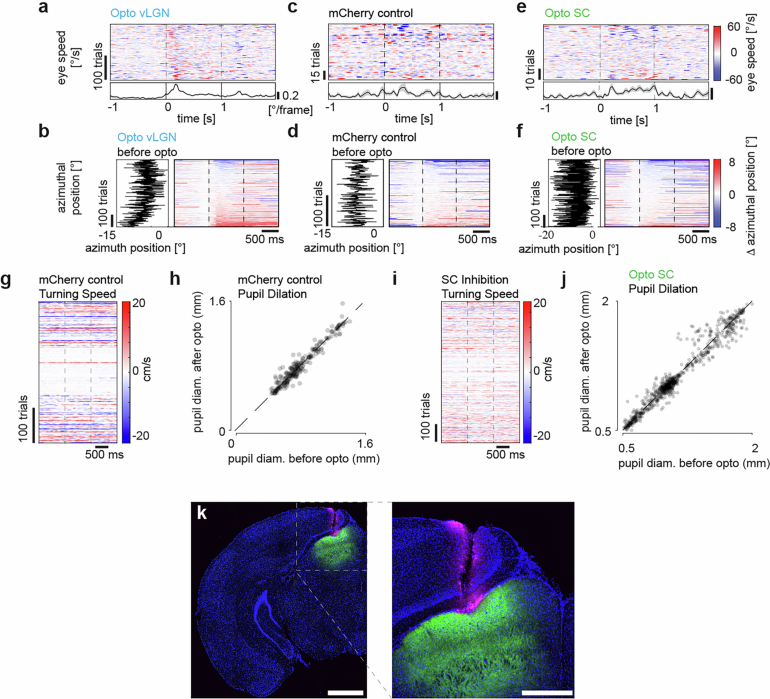
Optogenetic behavioral controls. **a**. Optogenetically induced saccadic eye movements sorted by mean angular speed during 0.25 s after the onset of optogenetic stimulation. Only the trials where the saccadic eye motion exceeded 14 °/s during the entire optogenetic stimulation are plotted (see also Fig. 4e). Gray dotted lines mark the start and end of the optogenetic stimulation. A clear velocity peak is visible at 150 ms post activation. Bottom: mean ± SEM (211 repetitions, 9 recordings, 3 animals). **b**. Starting eye position (left) and optogenetically induced saccade (right) sorted by displacement magnitude for ChR2 experiments (544 repetitions, 11 recordings, 4 animals). **c**. as (**a**) but for control mCherry experiments (62 repetitions, 3 recordings, 2 animals). **d**. as (**b**) but for control mCherry experiments (227 repetitions, 3 recordings, 2 animals). **e**. as (**a**) but for SC inhibition using panneuronal eNpHR3.0 (185 repetitions, 3 recordings, 3 animals). **f**. as (**b**) but for SC inhibition using panneuronal eNpHR3.0 (779 repetitions, 3 recordings, 3 animals). **g**. Optogenetically induced turning speed for control mice. Gray dotted lines mark the start and end of the optogenetic stimulation (229 repetitions, 3 recordings, 2 animals). **h**. Optogenetically induced pupillary dilation for control mice (227 repetitions, 3 recordings, 2 animals, Wilcoxon signed rank test, p = 0.88). **i**. as (**g**) but for SC inhibition experiments (780 repetitions, 3 recordings, 3 animals). **j**. Optogenetically induced pupillary dilation for SC inhibition mice (779 repetitions, 3 recordings, 3 animals, Wilcoxon signed rank test, p = 0.0002). **k**. Optic fiber track (magenta) and eNpHR3.0 expression (green) in the SC. Scale bars: left 1 mm, right 500 µm (n = 3 animals). Source data

Our results show that the vLGN acts as a feedback controller, fine-tuning sensory signals corrupted by motion blur (Figs. 1–5). However, anatomical projections to several motor-related areas suggest that the vLGN functions on a more global scale, coordinating visual and motor processes simultaneously. While motion blur corrections are relevant for any action and should be corrected in the periphery, other corrections may require specificity, especially if the actions occur on different time scales (for example, saccades are ballistic movements, and pupil dilation is continuous). Thus, we reasoned that optogenetic activation of vLGN pathways might reveal motor corrections related to the behaviors associated with its neuronal activity (Fig. 3). To test this, we analyzed the behavioral responses to vLGN activation in the previously recorded animals (Fig. 2), focusing on locomotion, saccadic eye movements and pupil size modulation (Fig. 6e–l). Upon optogenetic activation, mice robustly changed their gaze (Fig. 6f), which had a small corrective movement of ~3° on average and tended to increase in magnitude when the eye was further away from the central position before stimulation (Fig. 6f–h). We also observed a robust increase in pupil size upon optogenetic stimulation, independent of the initial dilation (Fig. 6f,i–j). Similarly, their walking direction and rotation of the spherical treadmill were strongly toward the side of optogenetic stimulation (Fig. 6k,l and Extended Data Fig. 8a,b). These behaviors do not appear to be mediated by the sSC, as optogenetic inhibition using panneuronal eNpHR3.0 of large parts of the sSC in control experiments showed no behavioral effects (Fig. 6e–l and Extended Data Fig. 8). These results show that beyond modulating visual signals, vLGN globally influence sensorimotor transformations, coordinating sensation and action.

### The vLGN controls corrective actions and arousal

**Fig. 7:**
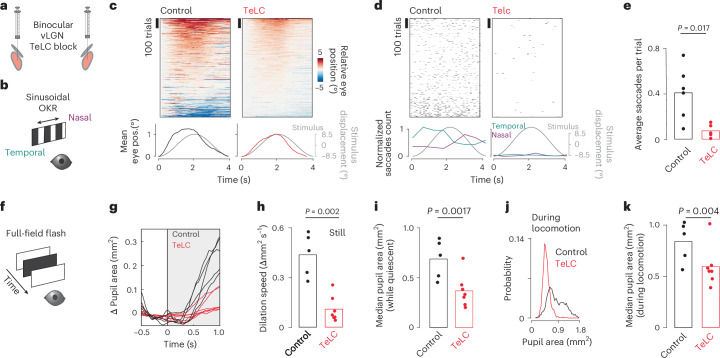
The vLGN activity is necessary for the coordination of behaviors and arousal. **a**, Schematic of bilateral viral TeLC expression in Gad2^+^ neurons in the vLGN/IGL complex. **b**, Schematic of optokinetic reflex paradigm. **c**, Top, sorted eye positions for control and TeLC animals in trials where animals were not locomoting. Each trial is zeroed to the starting position. Note the smooth appearance in TeLC animals compared with controls, indicating absence of saccades. Bottom, average eye position plotted next to the stimulus displacement. **d**, Top, saccadic events (displacements > 2.5° per frame), trial sorted as in **c**. Bottom, normalized saccadic counts for temporal and nasal saccades. Note: control animals correct the smooth optokinetic tracking with saccadic events, a behavior absent in TeLC animals. **e**, Average saccadic events per trial per session (Wilcoxon rank sum test, control: 5 animals, 6 sessions, TeLC, 6 animals, 7 sessions, same for **c** and **d**). **f**, Schematic of pupillary reflex paradigm. **g**, Average stimulus-evoked pupil dilation per animal (control 5 animals, TeLC 7 animals). **h**, Quantification of the stimulus-evoked dilation speed from **g** (Wilcoxon rank sum test, same for **i** and **k**). **i**, Median pupil area during still periods for control and TeLC animals. **j**, Pupil area distribution during running for control and TeLC animals. **k**, Median pupil area during running periods for control and TeLC animals. OKR, optokinetic reflex. Source data

**Fig. 8:**
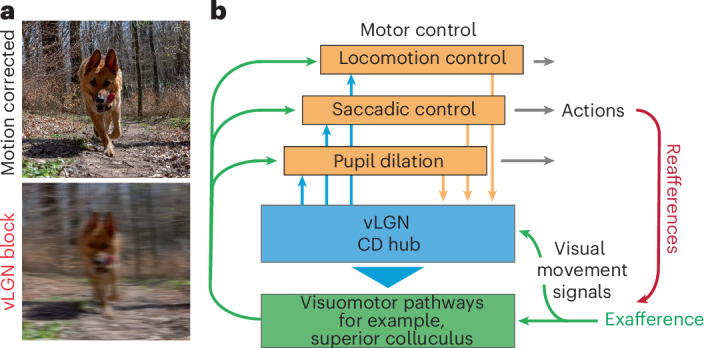
Summary of the vLGN function in coordinating sensation and action. **a**, Illustrative motion blur correction. **b**, Schematic of the vLGN hub-and-spoke feedback loops that coordinate vision and behavior.

Optogenetic vLGN activation can drive corrective movements consistent with its functional repertoire (Figs. 3 and 6), suggesting that the vLGN is part of a finely tuned feedback control system that adapts to the behavioral needs of the moment. To test the role of the vLGN as a global feedback controller that coordinates visual and behavioral transformations in parallel, we quantified fine-grained motor deficits in animals in which the vLGN was blocked by targeted bilateral TeLC expression (Fig. 7a). First, we tested whether vLGN-blocked animals would have deficits in visuomotor transformations, such as with the optokinetic reflex. For this, we used a sinusoidally moving random checker and measured tracking accuracy by eye movements^52^ (Fig. 7b). On average, control and vLGN-blocked animals could track the sinusoidal movement to a similar extent (Fig. 7c). However, while control animals showed a large number of saccades in the direction of stimulus motion, on average 0.4 saccades per trial, vLGN-blocked animals had essentially no saccades during nonrunning epochs (Fig. 7d,e). The functional interpretation of such corrective actions remains elusive, but they appear to reveal mechanisms required to improve the accuracy and stability of eye movements similar to those described in humans^53^. Next, we tested whether the sensory-evoked pupil constriction was affected by measuring the pupil constriction reflex in animals with TeLC-mediated vLGN block, compared with control animals (Fig. 7f). We observed a drastic decrease in the constriction rate in vLGN-blocked animals (Fig. 7g,h). Next, we tested whether pupil dynamics were altered in relation to locomotion state. During both stationary (Fig. 7i) and running periods (Fig. 7j,k), pupil sizes were significantly smaller in vLGN-blocked animals, never reaching a fully dilated state. These results emphasize that the fine control of visually driven and running/arousal-coupled oculomotor behavior requires signals relayed by the vLGN, and suggest that the vLGN synchronizes both visual processing and animal behavior in real-time (Fig. 8b).

## Discussion

All animals need mechanisms to distinguish sensory inputs caused by their own movements (reafferent) from those originating in the external world (exafferent) (Fig. 1a). In the visual system, this is supported by mechanisms starting at the retina^54,55^ and by ocular stabilization systems such as the vestibulo-ocular reflex^56–58^. However, despite this precisely tuned motor feedback system, some self-induced visual motions, such as those elicited during translational movements, cannot be fully stabilized. An additional corrective strategy involves CDs^1^—neural signals transmitting a copy of motor commands to sensory-processing regions, providing predictive information about expected sensory feedback. CD has been extensively studied in saccades, rapid eye movements that would temporarily blur visual perception. To counteract this perceptual blurring, a process called saccadic suppression has been found across species^59–64^. In primates, ascending pathways from the midbrain^6^ to the frontal eye field, a cortical structure involved in visual processing and oculomotor control^65^, mediate this process. In rodents, the lateral posterior nucleus, homologous to the pulvinar, has also been linked to correcting action-induced sensory signals, particularly during saccades^44,66^.

Beyond saccadic eye movements, reafference signals induced by self-motion influence visual processing in various ways. One approach is for different brain areas to independently generate CDs to compensate for different behaviors, although maintaining reliable sensory representation in a distributed system is challenging. Alternatively, a CD hub could pool all possible behavioral signals and modulate sensory signals according to the best overall estimate of the currently executed movement ensemble, ideally as early as possible. Here, we provide evidence for the latter by showing that a strong inhibitory projection from the vLGN (Fig. 1) modulates visual signals in the sSC (Fig. 2). We found that this inhibitory projection mediates a combination of visual and behavioral parameters (Fig. 3) that are distinct from direct visual inputs from the retina (Extended Data Fig. 3). In comparison, while retinal terminals show a variety of visual responses as previously shown^40^, vLGN terminals are mainly driven by high-frequency luminance modulation and front-to-back visual motion (Fig. 3), stimuli that occur during forward locomotion in natural environments. Actions such as saccades, locomotion or pupil dilation in visually homogeneous environments barely modulate retinal inputs, but strongly drive the vLGN projection. In addition, vLGN activity appears to relay direct copies of the motor action (Figs. 1 and 3), which would coincide with the arrival of visual inputs delayed by phototransduction. Accordingly, animals with a blocked vLGN showed saccade suppression deficits (Fig. 4), a computation that alleviates the visual blurring caused by different behaviors (Fig. 8a). Interestingly, such computations are achieved by largely preserving the temporal code of the first spike while reducing the total number of spikes (Figs. 2i–m and 4g–i), suggesting that the onset of visual signals remains informative and relevant irrespective of whether it is of reafferent or exafferent origin. While our data have been focused on the SC, anatomical evidence shows that other early sensory areas also receive direct inputs from the vLGN, for example, the lateral posterior nucleus and dLGN (Fig. 6), and thus are likely being similarly modulated. Consistently, vLGN-blocked animals could not discriminate depth via motion parallax (Fig. 5). Taken together, our results reveal a powerful hub for sensory modulation that is a critical first step in attenuating the impact of sensory stimuli during action.

In the sensory periphery, the vLGN appears to regulate sensory signals, allowing the brain to compare predicted sensory inputs that originate through behavior with actual sensory signals. Such a feedback loop would enable constant adjustments of visuomotor transformations in real-time^1,6^, and thus improve perception. However, our functional and anatomical data suggest a more nuanced view beyond the classical view of the CD. The vLGN simultaneously coordinates and fine-tunes multiple sensorimotor processes. This is evident from optogenetic activation of the vLGN, which induces changes in locomotor speed, saccadic eye movements and changes in pupil size (Fig. 6e–l). Accordingly, anatomical evidence shows that the vLGN projects to and influences other subcortical target areas known to strongly influence these behaviors, such as the red nucleus and the Edinger–Westphal nucleus, the latter via the olivary pretectal nucleus^48^ (Fig. 6a–d and Extended Data Fig. 6). Consistently, blocking vLGN output alters sensory-evoked and internally controlled saccadic and pupil dilation dynamics (Fig. 7).

Action-induced sensory signals, such as motion blur, are generated by various actions, from locomotion to head and saccadic eye movements. Thus, a common correction in early sensory areas, that is, SC, dLGN and lateral posterior nucleus (Figs. 1 and 6 and Extended Data Fig. 6), would be ideal. Our results support this by showing that a mixture of behavioral features, in most cases in a bouton-specific manner, are mapped to SC (Fig. 3i–m). However, behaviors can be highly variable in their kinetics and require specialized control systems, for example, comparing a slow pupil dilation with a ballistic saccade. Thus, the vLGN consists of a population of highly specialized feedback loops that operate in orchestration (Fig. 8b). This architecture facilitates both sensory adaptations that can be generalized across actions, such as motion blur compensation, and behavior-specific adaptations tailored to the dynamic properties of different actions. This is supported by vLGN single-cell sequencing data showing large neuronal diversity^43^, previous studies linking vLGN projections to divergent behaviors^15^, the diverse projections associated with specific behaviors (Fig. 6 and Extended Data Fig. 6) and the behavioral deficits when the vLGN is blocked (Fig. 7). As there are some discrepancies in the literature regarding the exact projections of the vLGN^16,29^, it is likely that these arise also from different neuronal populations with different projection patterns. Some will project mainly to the intermediate SC layers^16^, others more to the sSC layers^29^. In our study, we focused primarily on the sSC neurons, all of which receive strong inhibitory modulation from vLGN Gad2^+^ neurons (Fig. 1).

The vLGN is also known to relay visual information by receiving a variety of retinal inputs^33^. Consistent with previous findings, we show that vLGN neurons have large RFs, relaying changes in overall luminance^29,41^ (Fig. 3). These properties can sharpen RFs in the SC (Fig. 2), a computation that has been postulated to support visually guided approach behavior^29^. However, we also show that the strongest visual responses are due to high-frequency flicker and motion stimuli typically observed during forward translation (Fig. 3e–h). Such high-frequency and motion stimuli may occur when an animal runs under the canopy and may support other action cues conveyed by the vLGN. The exact computational role of such visuomotor interactions remains to be determined. Recently, the vLGN has also been implicated as an inhibitory switchboard for behavioral control^67^ and, via its projections to the SC and nucleus reuniens, serves as a key regulator for adjusting defensive behaviors according to previous experience and the level of perceived visual threat^15,16^. All of these findings suggest that CD signals are also modulated in a state-dependent manner, adding an additional layer of complexity to how animals process early sensory signals to adapt their perception to the demands of the moment.

It is worth noting that the vLGN is a neural structure present across amniotes, from reptiles to primates, including humans^20,68^. While this nucleus exhibits variations in subdivisions, cell groups and co-neurotransmitters, the vLGN shows a high conservation of gross connectivity^20^ and its main neurotransmitter, GABA. In macaques, the vLGN homolog (pregeniculate complex) is involved in visuo-oculomotor activity^69^, underscoring the conserved sensory-motor nature of the vLGN. Future comparative research, including humans, will be needed to unravel the specifics and extent of this CD motif across species.

While our results are consistent with the concept of CDs, it is worth noting that our study is far from a comprehensive understanding of the underlying computation. Because we do not know how visual signals are subsequently processed by the brain and interpreted by the organism, we cannot define how the vLGN enables the distinction between sensory experiences elicited by external stimuli and those elicited by one’s own actions. It is likely that these computations are much more complex. The vLGN has been shown to have a large diversity of inhibitory neurons^43^, but our current experimental capabilities treat them all as a homogeneous group. It is likely that some of the broad visuomotor functions reported here are distributed across multiple vLGN subcircuits (Fig. 8b). Thus, our global analysis of the vLGN is unlikely to reveal the underlying computational complexity required for proper sensation during actions.

In conclusion, the vLGN plays a fundamental role in the coordination of motor actions and visual processing, thereby maintaining perceptual stability. Our results underscore how closely vision and motion are linked to enable effective interaction with the sensory environment.

## Methods

### Animals

Animal protocols were reviewed by the institutional preclinical core facility at the Institute of Science and Technology Austria (ISTA). All breeding and experimentation were performed under a license approved by the Austrian Federal Ministry of Science and Research in accordance with the Austrian and EU animal laws (BMF-66.018/0017-WF/V/3b/2017). During the experimental phase, mice were housed individually in standard macrolon cages with red plastic houses, running wheels and enrichment consisting of wood chips and nesting material, on an inverted 12-h light cycle. Experiments were done during the dark phase of the light cycle.

For in vivo tracing (*n* = 15, 8 males, 7 females), ex vivo patch-clamp (*n* = 11, 7 males, 4 females), in vivo opto/electrophysiology (*n* = 9, 5 males, 4 females), in vivo vLGN terminal imaging (*n* = 6, 4 males, 2 females) and in vivo TeLC experiments (*n* = 12, 6 males, 6 females), Gad2-IRES-Cre (JAX, cat. no. 010802) mice, aged 8 weeks (5–12 weeks for vLGN bouton imaging) at viral injection, were used. For in vivo anterograde transsynaptic experiments, Ai75D (JAX, cat. no. 025106, *n* = 6, 4 males, 2 females) mice, aged 8 weeks at viral injection, were used. For in vivo retrograde transsynaptic experiments, NTSR1-GN209-Cre (MMRRC, cat. no. 030780, *n* = 4, 2 males, 2 females), GRP-KH288-cre (MMRRC, cat. no. 031183, *n* = 4, 2 males, 2 females) and Rorb-Cre (JAX, cat. no. 023526, *n* = 4, 2 males, 2 females) mice, aged 8 weeks at viral injection, were used. The mice for retinal terminal imaging experiments were C57BL/6J (JAX, cat. no. 000664; *n* = 5, 3 males, 2 females), aged 6–11 weeks at eye injection. Of those, three mice have been used to record previously published separate datasets^70^.

### Statistics and reproducibility

No statistical methods were used to pre-determine sample sizes but our sample sizes are similar to those reported in previous publications^15,16,29^. Extracellularly recorded units and imaged bouton regions of interest (ROIs) were selected or excluded based on quality and response criteria as described below. No other data points or animals were excluded in this study. For behavioral experiments, animals were randomly assigned to control and experimental groups and tested in random order. Experimenters were not blinded to the assignment of animals to experiments. However, the procedure for behavioral testing and data collection was automated and pooled for batch analysis. For all visual stimulation experiments, stimulus presentation was randomized.

### Viral vectors

Anterograde transsynaptic expression was done with AAV1-cre (AAV1.CamKII0.4.Cre.SV40, 7 × 10^12^ genome copies per milliliter, Addgene). Retrograde transsynaptic expression was performed with starter vector (AAV-DIO-Ef1a-TVA-FLAG-2A-N2C_G)^30^ and pseudotyped rabies vector (N2C(Enva)-EGFP, ~2–5 × 10^8^ genome copies per milliliter)^30^. Calcium indicator expression in vLGN/IGL neurons was achieved with AAV-hSynapsin1-FLEx-axon-GCaMP6s (1 × 10^12^ genome copies per milliliter, Addgene, cat. no. 112010-AAV5) for retinal expression AAV2.7M8-syn-GCaMP8m viral vectors (1 × 10^13^ genome copies per milliliter), generated at ISTA viral facility. TeLC viruses were generated using AAV5-hSyn-FLEX-TeLC-P2A-dTomato (Addgene, cat. no. 159102, 1 × 10^13^ genome copies per milliliter) at ISTA viral facility. ChR2 (AAV5-EF1a-doubleFloxed-hChR2(H134R)-EYFP-WPRE-HGHpa (cat. no. 20298-AAV5), 1 × 10^13^ genome copies per milliliter), mCherry control (AAV5-hSyn-DIO-mCherry (cat. no. 50459-AAV5), 7 × 10^12^ genome copies per milliliter) and eNpHR3.0 (AAV5-hSyn-eNpHR3.0-EYFP (cat. no. 26972-AAV5), 10^13^ genome copies per milliliter) viruses were purchased from Addgene.

### Stereotaxic viral injections

Anesthesia was induced with 3% isoflurane and intraperitoneal (i.p.) ketamine and xylazine (100 mg kg^−1^, 10 mg kg^−1^). As an analgesic, meloxicam (20 mg kg^−1^) was subcutaneously injected. Mice were placed in a stereotaxic apparatus (Kopf) and body temperature was controlled with a heating pad at 37 °C throughout the whole procedure. Stereotaxic target coordinates relative to bregma were: −2.3 mm anterior-posterior (AP), 2.5 mm medial-lateral (ML), 3.4 mm dorso-ventral (DV) for vLGN; −2.3 mm (AP), 2.3 mm (ML), 3.4 (DV) for medial thalamus; −3.8 mm (AP), 0.8 mm (ML), 1.3 mm (DV) for SC; and 4 mm (AP), 2.6 mm (ML), 0.5 mm (DV) for visual cortex injection. Glass electrodes were pulled with a one-stage puller (DMZ-Zeitz-Puller) to produce a tip opening ~30 μm. The pipette was filled with mineral oil, then attached to a Nanoliter 2010 (World Precision Instruments) and loaded with the respective vector. Pipettes were slowly lowered to the target region (vLGN 150 nl/300 nl, SC 200 nl/300 nl, primary visual cortex 60 nl) and the solution was injected at a rate of 45 nl min^−1^. Once the volume was delivered, pipettes remained in place for 15 min before being carefully withdrawn and the incision closed with VetBond (3M). For retrograde transsynaptic tracing, the pseudotyped rabies vector was injected 7 d after the first starter vector injection. Otherwise, animals were recovering and awaiting viral expression for at least 3 weeks, before further experiments were conducted. For vLGN terminal imaging experiments, in four of six mice viral infection was immediately followed by cranial window implantation in the same surgery. The remaining two mice were implanted 4 weeks after the injection surgery.

### In vitro electrophysiology

Mice were deeply anesthetized via i.p. injection of ketamine (95 mg kg^−1^) and xylazine (4.5 mg kg^−1^), followed by transcardial perfusion with ice-cold, oxygenated (95% O_2_, 5% CO_2_) artificial cerebrospinal fluid (ACSF) containing (in mM): 118 NaCl, 2.5 KCl, 1.25 NaH_2_PO_4_, 1.5 MgSO_4_, 1 CaCl_2_, 10 glucose, 3 myo-inositol, 30 sucrose, 30 NaHCO_3_; pH 7.4. The brain was rapidly excised and coronal sections of 300-µm thickness containing the SC were cut using a Linear-Pro7 vibratome (Dosaka). Slices were left to recover for 20 min at 35 °C, followed by a slow cool down to room temperature over 40–60 min. After recovery, one slice was transferred to the recording chamber (cat. no. RC-26GLP, Warner Instruments) and superfused with ACSF containing 2 mM CaCl_2_ at a rate of 3–4 ml min^−1^ at room temperature (21.0–23.0 °C). Glass pipettes (cat. no. B150-86-10, Sutter Instrument) with resistances of 3–4 MΩ were crafted using a P1000 horizontal pipette puller (Sutter Instrument) and filled with internal solution containing (in mM): 140 K-gluconate, 2 MgCl_2_, 2 MgATP, 0.2 NaGTP, 0.5 EGTA, 10 HEPES; pH 7.4 adjusted with KOH. Biocytin (0.2–0.3%) was added to the internal solution for post hoc morphological reconstruction. Electrical signals were acquired at 20–50 kHz and filtered at 4 kHz using a Multiclamp 700B amplifier (Molecular Devices) connected to a Digidata 1,440 A digitizer (Molecular Devices) with pClamp10 software (Molecular Devices). For optogenetically evoked inhibitory postsynaptic currents, neurons were held at −60 mV and blue light (*λ* = 465 nm, 10–20-mW cm^−2^ intensity, 5-ms pulse duration, 0.1–0.2-Hz stimulation frequency) was emitted through a mono fiber-optic cannula (5-mm length, fiber diameter 200 μm, total diameter 230 μm, Doric Lenses) connected to a PlexBright LED 644 (Plexon) with an optical patch cable (fiber diameter 200 μm, total diameter 230 μm, 0.48 numerical aperture (NA)). To block GABA_A_ receptors, ACSF containing 20 µM bicuculline was bath-applied for 20–30 s followed by immediate washout. Access resistance was constantly monitored between protocols, and recordings with access resistances exceeding 20 MΩ or with changes in access resistance or holding current of more than 20% were discarded. After recordings, the pipette was carefully withdrawn and the slice was transferred to 4% paraformaldehyde in PBS solution.

### Viral eye injections

For expression of calcium indicators in retinal neurons, C57BL/6J mice were anesthetized with ketamine/xylazine (100 mg kg^−1^, 10 mg kg^−1^) by i.p. injection. A small hole in the temporal eye, below the cornea, was cut with a 1/2-inch, 30-gauge needle. Subsequently, 1 μl of vitreous fluid was withdrawn and 1 μl of AAV2.7M8-syn-GCaMP8m viral vector solution was injected into the subretinal space with a Hamilton syringe and a 33-gauge blunt-ended needle. Mice were left to recover and viral expression was to commence for 2–4 weeks before implantation of the cranial window.

### Cranial window implantation surgery

For cranial window implantation, mice were injected with meloxicam (20 mg per kg body weight, subcutaneous (s.c.), 3.125 mg ml^−1^ solution) and dexamethasone (0.2 mg per kg body weight, i.p., 0.02 mg ml^−1^ solution). Anesthesia was induced by 2.5% isoflurane in oxygen in an anesthesia chamber and maintained at 0.7% to 1.2% in a stereotaxic device (Kopf), while body temperature was controlled by a heating pad to 37.5 °C. After exposing and cleaning the cranium, a 4-mm circular craniotomy was drilled above the left SC, the dura mater was removed and the left transverse sinus was sutured twice with 9-0 monofil surgical suture material (B. Braun) and cut between the sutures. Cortical areas covering the left SC were aspirated with a cell culture vacuum pump (Accuris), and a 3-mm circular coverslip, glued (Norland optical adhesives 61) to a stainless-steel conical ring, was inserted with the glass flush on the surface of the SC. After filling the surrounding cavity with Dura-Gel (Cambridge Neurotech), the insert was fixed in place with VetBond (3M). Finally, a custom-designed TiAl_6_V_4_ head-plate was affixed to the cranium by sequential application and curing of (1) All-in-One Optibond (Kerr), (2) Charisma Flow (Kulzer) and (3) Paladur (Kulzer). Mice were given 300 µl of saline and 20 mg per kg body weight meloxicam (s.c.) before removing them from the stereotaxic frame and letting them wake up, while keeping them warm on a heating pad. Further doses of 20 mg per kg body weight meloxicam (s.c.) and 0.2 mg per kg body weight i.p. dexamethasone were injected 24 h after the conclusion of the surgery. After the implantation surgery, mice were allowed to recover for at least 1 week.

### Setups for head-fixed in vivo recordings

For awake, behaving experiments, two similar setups were used, with the difference that one was coupled to a custom-built multiphoton setup, and the other allowed for silicon probe/neuropixels recordings. In short, mice were head-fixed while awake using a custom-manufactured clamp (for imaging: connected to a three-axis motorized stage (cat. no. 8MT167-25LS, Standa)) and could run freely on a custom-designed spherical treadmill (20-cm diameter). Running behavior was recorded by a pair of ADNS-3080 (iHaospace, Amazon) optical flow sensor modules, focused with 25-mm lenses (cat. no. AC127-025-AB-ML, Thorlabs) on a small patch at orthogonal locations of the Styrofoam ball and illuminated by an 850-nm light-emitting diode (LED). The alternating sensor readout was controlled at 50 frames per second by an Arduino Uno running custom scripts. The four signal channels from the sensor were linearly mapped to movement speed in the forward, sideways and rotational axes based on regular calibration with synchronous measurement of image translations and rotation at the ball’s apex. Eye and body movements were recorded at 50 frames per second with infrared illumination (850 nm) with a camera (cat. no. acA1920-150um, Basler) and an 18–108-mm macro zoom objective (MVL7000, Thorlabs) for multiphoton imaging or a fixed focal length objective for electrophysiology (Edmund Optics, *f* = 50 mm, cat. no. 59-873), pointed at the right side of the mouse via an infrared mirror. Eye position and saccades were determined post hoc as previously reported^70^, by first labeling eight points around the pupil with DeepLabCut^71^, which were fitted to an ellipse, and the center position was transformed to rotational coordinates. Fast eye position changes of more than 45° s^−1^ and at least 3° amplitude on a 0.7-s median filtered trace were defined as saccades. The ellipse area in mm^2^ was determined as pupil size.

Visual stimuli were projected by a modified LightCrafter (Texas Instruments) at 60 Hz (Multiphoton setup: DLP LightCrafter evaluation module; e-phys setup: DLP LightCrafter 4500, Texas Instruments), reflected by a quarter-sphere mirror (Modulor) below the mouse and presented on a custom-made spherical dome (80 cm in diameter) with the mouse’s head at its center. For imaging experiments, a double bandpass filter (387/480 HD Dualband Filter, Semrock) was positioned in front of the projector to minimize light contamination during imaging. In both setups, the blue LED in the projector was replaced by ultraviolet (cat. no. LZ1-00UB00-01U6, Osram) and, in addition, in the multiphoton setup, the green LED was replaced by a cyan LED (cat. no. LZ1-00DB00-0100, Osram) not to interfere with the calcium imaging wavelengths. The reflected red channel of the projector was used for synchronization and captured by a trans-impedance photo-amplifier (cat. no. PDA36A2, Thorlabs) and digitized. Stimuli were designed and presented with Psychtoolbox-3 (ref. ^72^), running on MATLAB (MathWorks) on Microsoft Windows 10 systems. Stimulus frames were morphed on the GPU using a customized projection map and an OpenGL shader to counteract the distortions resulting from the spherical mirror and dome. In both setups, the dome allows the presentation of mesopic stimuli from circa 100° on the left to circa 135° on the right in azimuth and from circa 50° below to circa 50° above the equator in elevation. In between dynamic stimuli presented in randomized order, the screen was set to a homogeneous gray (green and ultraviolet light) at scotopic level for at least 30 s. To determine behavioral coupling, these stimuli were interspersed with 5-min gray screens, that is, at visual baseline.

### In vivo electrophysiology and optogenetics

Gad2-Cre mice, previously injected with AAV5-EF1a-doubleFloxed-hChR2(H134R)-EYFP, were anesthetized with isoflurane (1–1.5% in oxygen 0.8 l min^−1^) and injected with meloxicam (20 mg kg^−1^, s.c.) and placed in the stereotaxic apparatus. The skull was exposed and the periosteum and connective tissue removed. Thin crossed grooves over the bone were cut to increase the contact surface using a scalpel. The skull was first covered with a thin layer of cyanoacrylate (VetBond, 3M), then Charisma Flow (Kulzer) that was blue-light-cured for 45 s, before securing a head-plate with SuperBond dental adhesive resin cement (Sun Medical). A tapered optic λ-fiber with an active zone of 0.5 mm (NA 0.39, Optogenix) was implanted using the same vLGN coordinates and craniotomy as the injection. The tip of the fiber was slowly lowered to a depth of 3.4 mm from the dorsal surface and cemented to the skull.

At 1 d before the recording session, mice were anesthetized with isoflurane (1–1.5% in oxygen 0.8 l min^−1^) and injected with meloxicam (20 mg kg^−1^, s.c.). A small craniotomy was made in the rostral skull (bregma: 0.5 mm AP, 2 mm ML) for implanting an inverted gold pin as a reference electrode. A second rectangular craniotomy was made over the cortex/SC region (bregma: −3.5–3.8 mm AP, 0.5–1 mm ML), leaving the dura mater intact. The window was covered with silicone elastomer (Kwik-Cast, World Precision Instruments). The next day, Kwik-Cast was removed and the well around the craniotomy was constantly filled with ACSF throughout the whole recording session. Extracellular recordings were obtained using a single shank acute linear 32-channel silicon probe (ASSY-37 H4 with probe tip sharpening, Cambridge Neurotech) connected to an RHD 32-channel amplifier board and RHD2000 USB Interface Board (Intan Technologies) and Neuropixels 2.0 multishank probes (IMEC), using a Neuropixels data-acquisition system (see www.neuropixels.org for more detail). Before recording, the tip of the electrode was coated with DiI (Invitrogen) to allow post hoc recording site location. To access the sSC, the probe was slowly inserted through the cortex at a speed of 1 μm s^−1^ to a depth of ~1.7 mm using a stable micromanipulator (Luigs & Neumann Motorized). The electrode was left in place for 30 min before starting to record. Data were sampled at 20 kHz using Labview 2017 (National Instruments). Spike-sorting was performed with Kilosort 2 (https://github.com/cortex-lab/Kilosort)^73^. The automatic template of Kilosort 2 was manually curated on Phy2. The 473-nm laser (cat. no. SDL-473-XXXSFL-RA, Shanghai Dream Laser Technology) bursts for optogenetics were generated in Arduino Due (www.arduino.cc) in pulses of 40 Hz with an approximate power at the fiber tip of 2.5 mW mm^−2^.

### Visual local flash and optogenetics

Before starting the experiment, the visual field was scanned with a dark disk with 10° radius to determine the approximate location of RFs. We used this location to present a white or dark disk of the same radius. The visual local flash was interleaved in time with the laser burst of the same length, stimulating optogenetically vLGN. The duration of the laser and visual stimulation was 200 ms (Fig. 2e–g,i) or 1 s (Fig. 2h and Extended Data Fig. 8a–f). Laser burst onsets relative to the onset of the visual flash were randomized and varied in 13 increments. The start of the burst *i* was set to −1.5 × *t*_flash_ + *i* × *t*_flash_/4 for *i* in [0, 12], where *t*_flash_ was the duration of the flash.

### CSD analysis

To confirm the location of the silicone probe during the in vivo recordings, CSD analysis^74,75^ was applied. For this analysis, local flash stimuli that were at least 0.5 s after the laser burst were used. For each such repetition of the flash, CSD profile^75^ was computed on the raw voltage recorded values in the interval [−0.1, 0.2] s around the flash onset and averaged over multiple repetitions. The channel of the silicone probe corresponding to the current sink is defined as the channel where the current flow is the smallest. The closest channel above with positive current flow is the source. The depth of the source channel was set to 300 μm; the depth of the remaining channels was derived relative to the source using the 25-μm spacing between the channels. The response magnitude (Fig. 2f) was computed as the variance of the CSD profile of each channel across time. The normalized response (Fig. 2g) is the variance across all channels after the variance before the onset of the flash was subtracted and normalized to the maximum. The same procedure was applied to compute the CSD analyses around laser bursts, but selecting laser burst onsets that were at least 0.5 s after a visual flash.

### Neuronal responses

Both the zeta-test^76^ and a permutation test^77^ with subsampling were used to identify units that were responsive (*P* < 0.01) to the visual or optogenetic stimulation. The two tests detect complimentary response types: the zeta-test identifies event-locked responses, whereas the permutation test captures changes in the mean firing rate, including tonic changes of the firing rates, such as in the case of optogenetic stimulation of vLGN/IGL complex. For the permutation tests, the firing rate during the stimulus was compared with the baseline firing rate, estimated from random samples of 0.2-s intervals before the stimulus. Units were defined as visually responsive if they had either ON or OFF responses to the flash within 0.2 s after the start/end of the flash. A unit was considered optogenetically responsive if it exhibited a change of the spontaneous firing rate during 0.2 s after the start or end of the laser burst or if its visual ON/OFF responses were altered in the presence of optogenetic stimulation (permutation test). For analyses in Fig. 2h–l, only units responsive to both the visual flash and optogenetic stimulation were selected. To compute responses to visual flashes, optogenetic stimulation and both of the above (Fig. 2h–j and Extended Data Fig. 2b–d,f), we used the trials where the visual flash preceded optogenetic stimulation (visual responses, Fig. 2h and Extended Data Fig. 2b), or vice versa (optogenetic responses, Fig. 2h and Extended Data Fig. 2b), or where visual and optogenetic stimulation overlapped (visual and optogenetic responses, Fig. 2h and Extended Data Fig. 2b). In Fig. 2l and Extended Data Fig. 2e, the mean response of all units per laser offset was computed after normalizing the responses of each unit to their maximum across all laser offsets. To analyze RFs (Fig. 2m), a vertical bar (size 2° and 8°) was presented at a random horizontal position on the screen, and the location of the bar was updated with the frequency 15 Hz. At the same time, the pulses of optogenetic stimulation of LGN/IGL complex happened with the frequency of 1.4 Hz and pulse duration of 0.1 s, and this short duration of optogenetic pulses did not cause pupil dilation. To avoid the effect of rebound spiking, the spikes within 0.1 s after optogenetic pulses were removed from the analysis. We subsampled the spikes so that the numbers of spikes in conditions with and without optogenetic stimulation matched for each unit. To reconstruct RFs, we averaged the frames presented during [−0.5, 0.08] s around each spike, separating conditions into groups with and without optogenetic stimulation at the time of the spike. For further analysis, we excluded the units with noisy RF in either of the two conditions. For this, signal-to-noise ratio (SNR) was computed as the ratio of the variance of the RF in the time interval [*T* − 1, *T* + 1], to the variance outside of this interval, where *T* is the time of the maximal RF variance. The threshold for SNR was set to the 80th percentile of SNR of all units, at the value 3.44. The horizontal profile of an RF (Fig. 2m) was computed as the mean of three frames around *T*. We fit the one-dimensional Gaussian function (Fig. 2m) *g*(*x*) = *A* × exp((*x* − *m*)^2^/(2*w*^2^)) + *b*, where the parameters *A*, *m*, *w* and *b* are the amplitude, mean, width and the baseline. The fitting was done using the lsqcurvefit MATLAB function. The width of the RF (Fig. 5n) was estimated using the fitted parameter *w*.

### Optogenetically triggered behavior analysis

For vLGN, tapered optic λ-fibers with an active zone of 0.5 mm (NA 0.39, Optogenix) were implanted using the same vLGN coordinates as vector injections. For SC, optic fibers (400-μm diameter, NA 0.39, ThorLabs) were implanted at 1,000 μm from the pial surface using the same AP and ML coordinates as vector injections. Both types of fiber were fixed using light-curing glue (Optibond Universal, Kerr Dental) and dental cement (SuperBond C&B Kit, Hentschel-Dental).

To analyze optogenetically triggered behaviors, only 1-s optogenetic stimulation pulses, where the offset preceded the visual flash, were included. To determine turning speed (Fig. 6k), mean speed in a window of 0.25 s before stimulation onset was subtracted and trials sorted by mean speed in a 0.5-s window after stimulation onset, for visualization. Maximum turning speed (Fig. 6l) is the maximum within 0.25 s after the laser onset. Change of azimuthal pupil position (Δaz) was normalized and sorted using the same windows, defining starting position as the mean pupil azimuth between 0.25 s before and at laser onset (Extended Data Fig. 8b,d,f). Pupil velocity (Extended Data Fig. 8a,c,e) was computed as a central difference of sequential pupil azimuth values *v*_*t*_ = (az_*t* + 1_ − az_*t* − 1_)/2. Maximum pupil velocity (Fig. 6h) is the maximum of the velocity profile of each trial in the 0.25 s after the laser onset, and the mean of each group was computed from the trials in Extended Data Fig. 8a,c,e. Change in pupil azimuth (Fig. 6g) was defined as Δaz = az_*T* − *w*/2_ *−* az_*T* + *w*/2_, with the location *T* and width *w* of the maximum peak in the pupil velocity profile computed using the MATLAB function findpeaks. To determine changes of pupil size (Fig. 6i–j and Extended Data Fig. 8h,j), mean pupil diameter was computed during 0.25 s before and after laser offset; the difference between the two conditions was estimated using the Wilcoxon signed rank test (*P* = 10^−74^). Fig. 6j shows the pupil changes for all instances for the three experimental conditions in Fig. 6i and Extended Data Fig. 8h,j.

### In vivo vLGN terminal imaging

Two-photon terminal imaging in SC was performed using a custom build system, controlled by ScanImage (Vidrio Technologies) on MATLAB 2020b (MathWorks) using a pulsed Ti:Sapphire laser (Mai-Tai DeepSee, Spectra-Physics) set at wavelengths between 920 and 950 nm. The beam was expanded to underfill the back-aperture of the objective (×16, 0.8-NA water-immersion, Nikon) and scanned through the tissue by a galvanometric-resonant (8 kHz) mirror combination (Cambridge Scientific) and a piezo actuator (cat. no. P-725.4CA, Physik Instrumente) controlling the objective. Emission light was measured with GaAsP photomultiplier tubes (cat. no. H10770B-40, Hamamatsu) following collection by a dichroic mirror (cat. no. FF775-Di01, Semrock) and channel splitting (580-nm long-pass, cat. no. FF580-FDi01, Semrock) as well as filtering (green: cat. no. FF03-525/50; red: cat. no. FF01-641/75; Semrock). The signals were then amplified by a TIA60 amplifier (Thorlabs) and digitized with a PXI system (PXIe-7961R NI FlexRIO FPGA, NI 5734 16-bit, National Instruments). Average laser output power at the objective ranged from 38 to 125 mW (median of 75 mW). A field of view of 0.13–1.85 mm^2^ (median of 0.77 mm^2^) was imaged over 3–12 planes (median of 6 planes) with a plane distance of 10–45 µm (median of 28 µm), at a pixel size of 0.6–1.9 µm (median of 1.3 µm) and a volume rate of 4.2–9.5 Hz (median of 4.8 Hz). The field of view varied between recordings, ranging from 0.2 to 1.8 mm^2^ (median = 0.7 mm^2^) of the SC surface for vLGN/IGL terminal imaging and from 0.1 to 1.6 mm^2^ (median = 0.7 mm^2^) for retinal bouton imaging. Each mouse was recorded in 1–9 (median of 7) imaging sessions on different days. In a subset of recordings (*n* = 15) in separate imaging sessions, the absence of substantial *z*-motion was verified by injecting 40 µl of Texas Red dextran (3,000 MW, 14.3 mg ml^−1^, diluted in saline, Thermo Fisher) subcutaneously and imaging brightly red-labeled blood vessels at 980 nm (ref. ^78^).

### Visual stimuli for in vivo terminal response mapping

To measure sensitivity to luminance dynamics, repeated sequences of luminance chirps, as reported previously^70^, were used. The stimulus started at gray level, followed by a 1-s bright step and sinusoidal luminance changes over 8 s each, first with increasing amplitude at 2 Hz and then fixed full amplitude but frequency modulated (0 to 8 Hz). For determining direction selectivity, sinusoidal gratings of 0.1 cycles per degree spatial frequency, and 2 cycles per second temporal frequency, were presented at full contrast moving in 8 or 16 directions in randomized order. Gratings were presented stationarily for 3 s and then moved for 7 s in the current direction. To test for retinotopy, a dark bar with length spanning the screen and width of 25° was moved over gray background at 22.5° s^−1^ in 8 directions perpendicular to the bar orientation for 7 s with 3-s interval between presentations. Full-field flash responses were determined by presenting either dark or white 1-s full-field flashes from gray baseline at a pseudorandom interval of 5 to 10 s. Pseudosaccade stimuli consisted of vertical gratings with 0.08 to 0.25 cycles per degree (cpd) spatial frequency, or random checkerboard patterns with 4° to 12° visual angle checker size, which were presented on the screen. At pseudorandom intervals between 3 and 6 s, the full screen texture moved in a random horizontal direction over 0.08 s by a median of 5° (3° to 30°) visual angle. The distribution of such pseudosaccadic image shifts approximately matched those from actual saccades, as determined from head-fixed population spontaneous saccade statistics.

### In vivo axonal terminal imaging analysis

Imaging data were motion corrected and ROI segmented with suite2p (v.0.10.0)^79^ followed by a manual curation step based on morphological and activity shape. Note that multiple axonal ROIs can originate from the same neuron. Further analysis was performed in MATLAB (MathWorks). d*F*/*F*_0_, where d*F* is the fluorescence change over time and *F*_0_ the baseline fluorescence, was estimated as done previously^70,80^, by subtracting neuropil contamination with a factor of 0.5, defining *F*_0_ baseline as the 8th percentile of a moving window of 15 s (ref. ^81^) and finally subtracting and then dividing the fluorescence trace by the median of the same 15-s window. The fluorescence SNR was defined for each ROI by dividing the 99th percentile of the d*F*/*F*_0_ trace (‘signal’) by the standard deviation of its negative values after baseline correction (‘noise’). Only axonal segments with a fluorescence SNR ≥ 5 were included in further analysis.

To estimate modulation by visual and behavioral stimuli, a suite of stimuli (full-field flashes, frequency modulation, moving bar, moving full-field grating) was presented and a range of behaviors (locomotion, pupil size, saccades) were sampled. Modulation indices were computed by (*F*_response_ − *F*_baseline_)/(*F*_response_ + *F*_baseline_), *F* being the average d*F*/*F*_0_ value in a baseline or response window, respectively. For moving bar and grating stimuli, the baseline window was defined as [1.5, 0.1] s before movement start, and the response window from 0.25 s after stimulus start until the end of the stimulus. For full-field luminance modulation, baseline was between 1.3 s before and until frequency modulation start, and response was the time of frequency modulation presentation. For 0.5-s black or white full-field flashes, baseline window was 1 s before flash onset, and response window was 1 s following flash onset, including OFF responses. For saccades and locomotion onset analysis (Fig. 3k), [1.5, 0.5] s before respective onset was used as baseline and [0, 1] s following onset was used as response window. Significance of modulation was determined by two-sided Wilcoxon signed rank tests. Significance of correlations was determined by 5,000 repeats of randomly shifting the behavioral trace between 120 s and 1,200 s and computing correlations for shuffled datasets. Significance was then determined by calculating the proportion of shuffles with more extreme correlation values than the actual data. Boutons from either vLGN or retina were included in further analysis if they showed Bonferroni-corrected significant (*P* < 0.01) modulation or correlation to at least one of eight tested conditions. Note that not all recordings included all stimulus sections. These inclusion criteria removed 102,833 of 322,854 vLGNs and 22,646 of 101,376 RGCs over all recorded boutons from further analysis (Figs. 3 and 5e–h and Extended Data Figs. 3 and 4). To illustrate the diversity of responses to ‘chirp’ stimuli (Fig. 3e and Extended Data Fig. 3e), only boutons with an SNR > 0.35 are shown^70^. SNR was calculated as previously reported as the temporal variance of the mean across trials divided by the mean of the temporal variance of each trial. To illustrate the behavioral modulation of vLGN boutons (Fig. 3k), only boutons with at least ten trials in each condition (saccades, locomotion, pupil) are shown.

For pseudosaccade analyses (Fig. 5e–h), the baseline window was defined as [0.5, 0.1] s before and the response window as [0, 0.5] s following the pseudosaccade or saccade. Only saccadic events separated by at least 0.75 s from other saccadic events were included to avoid cross-contamination. Population synchrony (Extended Data Fig. 4a) was determined as variance of the population mean divided by the population mean of individual bouton variance. Similarly, individual bouton variance explained (Extended Data Fig. 4b) was determined by *z*-scoring bouton activity, and computing remaining variance after subtracting population mean (var_exp_ = 1 −var[act − <act>_population_]_*t*_, with act being *z*-scored bouton activity). To determine direction selectivity and preferred directions, 1 − circular variance and vector sums were used^82^. To demonstrate preferred direction distribution (Fig. 3f and Extended Data Fig. 3f), only boutons with significant direction tuning, *P* < 0.01 (10,000-fold shuffled direction label test), were plotted. To plot retinotopic alignment of bouton responses (Fig. 3g and Extended Data Fig. 3g), mean bouton responses to dark bars moving in nasal-temporal direction were determined. The centroids were projected into a set of one-dimensional axes, rotated at angles from 0° to 180° with the increment of 5° and binned at 20 µm. The responses of boutons within each bin were averaged. The axis that yielded the maximal correlation of the binned response peak-latency with the horizontal position of the bar was used for the alignment and the corresponding binned responses are shown in Fig. 3g and Extended Data Fig. 3g. To determine cross-correlation timing (Extended Data Fig. 4d,e), lag time of cross-correlation maximum was determined and the bouton included if peak lag was within [−3, 3] s and *P* < 0.01 (random shift test, see above). To disentangle independent locomotion and pupil size contributions to bouton correlations (Extended Data Fig. 4f–i), correlations to pupil size were separately computed for stationary periods (0.25-s window median filtered locomotion speed <1 cm s^−1^). Due to large sample sizes, comparisons between bouton populations yield arbitrarily low *P* values. In these cases, mean and standard deviation of the difference are reported alongside.

### Visual cliff setup

The visual cliff paradigm was performed in a black walled 50 × 50-cm^2^ acrylic box with 80-cm height, covered with transparent (5-mm thickness) and surrounded by black acrylic walls (~25-cm height). The illusory platform (25 × 25 cm^2^) was created by gluing a paper-printed checkerboard pattern to the bottom and adding a matching black acrylic border (width: 1 cm, height: 0.5 cm) to the surface of the transparent acrylic surface. Interior walls beneath the transparent surface as well as the box floor were covered with a high-contrast checkerboard pattern (2.5 × 2.5-cm^2^ black and white squares) so that all edges were aligned. At 1 d before experiments, mice were shortly anesthetized with isoflurane (5%) and vibrissae clipped next to the mystacial pad with surgical scissors. Once recovered, mice were returned to the maintenance cage*.* A camera (Basler, cat. no. acA1920-150um) with a fixed focal length objective (Edmund Optics, *f* = 50 mm, cat. no. 59-873) was located above the middle point of the arena to cover all movements. Camera control and recordings were obtained using a custom-made script in Python. Each mouse was recorded for 30 min while freely roaming in the arena, only the first 10 min of which were included in further analysis.

### Visual cliff analysis

The head and body were tracked using a custom-trained network in DeepLabCut^71^. Ear tag labels were used for trajectory analysis as they were the most reliable. Video frames were cropped to the arena size and scaled to 1,000 × 1,000 pixels for consistency. Platform area was defined as the bottom-left 25-cm quadrant of the arena, and the remainder as cliff area. The cliff avoidance index (Fig. 5d,e and Extended Data Fig. 5h,i) was computed as AI = (*t*_platform_ − *t*_cliff_)/(*t*_platform_ + *t*_cliff_), where *t* is the time spent in the platform or cliff normalized by the area of these regions. To compute the avoidance index, a 10-cm strip along the walls was excluded (Fig. 5b). Aborted exits (Fig. 5f,g and Extended Data Fig. 5j) were counted when a mouse crossed the platform boundary from inside the platform but then reversed the direction of movement normal to the platform boundary. Aborted exit trajectories were extracted for 4 s around these timepoints and multiple aborted exits removed.

### vLGN inactivation physiology

To quantify responses to saccades (Fig. 4g–i), the recordings during oscillating random checker stimulus were used. A random checker pattern (8° per checker) was oscillating sinusoidally by 17° in the horizontal direction for 450 cycles per recording, analogous to previous reports^52^. Up to two sessions were recorded per mouse; however, due to synchronization problems, some recordings had to be discarded. Only the first available recording per animal was used for the analysis. Saccades were identified as described above. Zeta-test^76^ was used to identify saccade-responsive neurons. To test optokinetic reflexes (Fig. 7a–e), the same stimulus was used. The starting pupil position per cycle was subtracted from the pupil position traces (Fig. 7c). Saccadic events (Fig. 7d,e) were identified as pupil displacements above 2.5° per frame. To determine luminance change responses, 1-s full-field flashes of three different intensities (low, medium and high) were presented in a random order. Pupil size response speed (Fig. 7g,h) was determined by estimating the slope of a linear fit to the relative pupil area change (baseline subtracted, fitted to the pupil values 0.2 s after flash onset). Quiescent and locomoting states (Fig. 7i–k) were identified as forward locomoting speed below and above 5 cm s^−1^. Zeta-test^76^ was done to detect visually responsive neurons, and peristimulus time histogram for white and black flashes was computed (Extended Data Fig. 5d–i).

### Euthanasia and histology

Mice were dosed with a 750–1,000 mg kg^−1^ mixture of ketamine/xylazine and transcardially perfused with PBS, followed by ice-cold 4% paraformaldehyde in PBS. Brains were carefully extracted and post-fixed overnight, cryoprotected with sucrose 30% and sectioned at 60 μm using a sliding microtome (Leica, cat. no. SM2010). Sections were collected in three series. The first series was used for signal amplification of the respective vector. Briefly, sections were incubated with PBST (Triton 0.3%) solution containing 5% donkey normal serum and one or more antibodies (goat Anti GFP, cat. no. ab6673, Abcam, diluted 1:2,000; rabbit anti-RFP, cat. no. 600-401-379, Rockland, diluted 1:1,000) overnight at 4 °C, followed by secondary fluorescent antibodies (Donkey anti-goat-488, cat. no. ab150129, Abcam, diluted 1:1,000; Donkey anti-rabbit-594, cat. no. R37119, Thermo Fisher, diluted 1:1,000) at room temperature for 1 h. Sections were mounted on slides and coverslips with custom-made mowiol.

### Confocal microscopy

Brain sections were imaged with a Nikon CSU-W1 spinning disk confocal microscope. All images were processed with FIJI (ImageJ).

### Statistics

Analyses were performed in custom-written MATLAB (MathWorks) and Python scripts. Nonparametric tests used are defined in the figure legends. All statistical tests are reported in the text and appropriate figure legends (*P < 0.05, **P < 0.01, ***P < 0.001). In bar plots the mean ± s.e.m. are shown, unless otherwise stated.

### Reporting summary

Further information on research design is available in the Nature Portfolio Reporting Summary linked to this article.

## Online content

Any methods, additional references, Nature Portfolio reporting summaries, source data, extended data, supplementary information, acknowledgements, peer review information; details of author contributions and competing interests; and statements of data and code availability are available at 10.1038/s41593-025-01874-w.

## Supplementary information

Reporting Summary

## Source data

Source Data Figs. 1–7 and Extended Data Figs. 2–5 and 8 Vega_Zuniga_Fig1_SourceData.xlsx in zip file. Compendium of all data shown in the figures. If the data were too large, there is a direct reference to the ISTA repository. Vega_Zuniga_Fig2_SourceData.xlsx in zip file. Compendium of all data shown in the figures. If the data were too large, there is a direct reference to the ISTA repository. Vega_Zuniga_Fig3_SourceData.xlsx in zip file. Compendium of all data shown in the figures. If the data were too large, there is a direct reference to the ISTA repository. Vega_Zuniga_Fig4_SourceData.xlsx in zip file. Compendium of all data shown in the figures. If the data were too large, there is a direct reference to the ISTA repository. Vega_Zuniga_Fig5_SourceData.xlsx in zip file. Compendium of all data shown in the figures. If the data were too large, there is a direct reference to the ISTA repository. Vega_Zuniga_Fig6_SourceData.xlsx in zip file. Compendium of all data shown in the figures. If the data were too large, there is a direct reference to the ISTA repository. Vega_Zuniga_Fig7_SourceData.xlsx in zip file. Compendium of all data shown in the figures. If the data were too large, there is a direct reference to the ISTA repository. Vega_Zuniga_EDFig2_SourceData.xlsx in zip file. Statistical source data. Vega_Zuniga_EDFig3_SourceData.xlsx in zip file. Statistical source data. Vega_Zuniga_EDFig4_SourceData.xlsx in zip file. Statistical source data. Vega_Zuniga_EDFig5_SourceData.xlsx in zip file. Statistical source data. Vega_Zuniga_EDFig8_SourceData.xlsx in zip file. Statistical source data.

## Data Availability

Data used in the analysis can be found at ISTA data repository: 10.15479/AT:ISTA:18579. Microscopy data reported in this paper will be shared by the lead contact upon request. Source data are provided with this paper.

## Code availability

Code used to generate the results is available at GitHub: https://github.com/joesch-lab/vLGN_SC.
