# Supplementary material for: A thalamic hub-and-spoke network enables visual perception during action by coordinating visuomotor dynamics
Source: Nat Neurosci. 2025 Feb 10;28(3):627–39. doi: 10.1038/s41593-025-01874-w (PMC11893466; doi:10.1038/s41593-025-01874-w)
Supplement: Supplementary file 1 — Reporting Summary [file 41593_2025_1874_MOESM1_ESM.pdf]

nature portfolio

Corresponding author(s): Maximilian Jösch, Tomas Vega-Zuniga

Last updated by author(s): Dec 9, 2024

Reporting Summary

Nature Portfolio wishes to improve the reproducibility of the work that we publish. This form provides structure for consistency and transparency in reporting. For further information on Nature Portfolio policies, see our Editorial Policies and the Editorial Policy Checklist.

Statistics

For all statistical analyses, confirm that the following items are present in the figure legend, table legend, main text, or Methods section.

n/a	Confirmed	
☐	☒	The exact sample size (*n*) for each experimental group/condition, given as a discrete number and unit of measurement
☐	☒	A statement on whether measurements were taken from distinct samples or whether the same sample was measured repeatedly
☐	☒	The statistical test(s) used AND whether they are one- or two-sided<br>*Only common tests should be described solely by name; describe more complex techniques in the Methods section.*
☐	☒	A description of all covariates tested
☐	☒	A description of any assumptions or corrections, such as tests of normality and adjustment for multiple comparisons
☐	☒	A full description of the statistical parameters including central tendency (e.g. means) or other basic estimates (e.g. regression coefficient) AND variation (e.g. standard deviation) or associated estimates of uncertainty (e.g. confidence intervals)
☐	☒	For null hypothesis testing, the test statistic (e.g. *F*, *t*, *r*) with confidence intervals, effect sizes, degrees of freedom and *P* value noted<br>*Give P values as exact values whenever suitable.*
☒	☐	For Bayesian analysis, information on the choice of priors and Markov chain Monte Carlo settings
☒	☐	For hierarchical and complex designs, identification of the appropriate level for tests and full reporting of outcomes
☐	☒	Estimates of effect sizes (e.g. Cohen's *d*, Pearson's *r*), indicating how they were calculated

Our web collection on statistics for biologists contains articles on many of the points above.

Software and code

Policy information about availability of computer code

Data collection	Behavioral data was collected using Labview 2017 (National Instruments) or Pylon (8.0.1, BASLER AG) recording software and analyzed via custom-made Python 3.7, running DeepLabCut (2.3.5) and Matlab 2020b scripts. 2P imaging data was recorded using ScanImage 2020 (Vidrio Technologies) on MATLAB 2020b (MathWorks), Electrophysiological data was acquired using custom software written in LabView 2017 (National Instruments) from Cambridge Neurotech probes or Neuropixels system (Neuropixels.org) for Neuropixel recordings, and stimuli were generated using Psychtoolbox-3 running on Matlab 2020b.<br>Microscopy images were processed in Fiji2 (2.14.0/1.54f).
Data analysis	Data analyses performed using custom code written in Python and Matlab and is available at GitHub: https://github.com/joesch-lab/vLGN_SC

For manuscripts utilizing custom algorithms or software that are central to the research but not yet described in published literature, software must be made available to editors and reviewers. We strongly encourage code deposition in a community repository (e.g. GitHub). See the Nature Portfolio guidelines for submitting code & software for further information.

Data

Policy information about availability of data

All manuscripts must include a data availability statement. This statement should provide the following information, where applicable:

- Accession codes, unique identifiers, or web links for publicly available datasets
- A description of any restrictions on data availability
- For clinical datasets or third party data, please ensure that the statement adheres to our policy

Data used in the analysis will be uploaded to ISTA data repository ISTA data repository DOI: 10.15479/AT:ISTA:18579. Microscopy data reported in this paper will be shared by the lead contact upon request.

Research involving human participants, their data, or biological material

Policy information about studies with human participants or human data. See also policy information about sex, gender (identity/presentation), and sexual orientation and race, ethnicity and racism.

Reporting on sex and gender	N/A
Reporting on race, ethnicity, or other socially relevant groupings	N/A
Population characteristics	N/A
Recruitment	N/A
Ethics oversight	N/A

Note that full information on the approval of the study protocol must also be provided in the manuscript.

Field-specific reporting

Please select the one below that is the best fit for your research. If you are not sure, read the appropriate sections before making your selection.

☒ Life sciences ☐ Behavioural & social sciences ☐ Ecological, evolutionary & environmental sciences

For a reference copy of the document with all sections, see nature.com/documents/nr-reporting-summary-flat.pdf

Life sciences study design

All studies must disclose on these points even when the disclosure is negative.

Sample size	No calculation was performed to predetermine sample sizes. Sample sizes were selected based on related research and literature. Sample size for each experiment is described in the figure legend. The number of individuals per experiment was sufficient to perform non-parametric tests for all experimental and control groups.
Data exclusions	Only animals were excluded if there was mistargeting of viral injection
Replication	All experiments
Randomization	Animals were randomly assigned to control and experimental groups, and tested in random order.
Blinding	The experimenters were not blinded to the allocation of animals to experiments. However, the procedure of behavioral testing and data collection was automatically controlled by the computer software, and the experimenters were blind to the order of visual stimulation. In addition, when analyzing the behavioral data, the investigators were blind to the experimental conditions because data obtained under different conditions were pooled for automatic batch analysis by the computer software.

Reporting for specific materials, systems and methods

We require information from authors about some types of materials, experimental systems and methods used in many studies. Here, indicate whether each material, system or method listed is relevant to your study. If you are not sure if a list item applies to your research, read the appropriate section before selecting a response.

Materials & experimental systems

n/a	Involved in the study	
☐	☒	Antibodies
☒	☐	Eukaryotic cell lines
☒	☐	Palaeontology and archaeology
☐	☒	Animals and other organisms
☒	☐	Clinical data
☒	☐	Dual use research of concern
☒	☐	Plants

Methods

n/a	Involved in the study	
☒	☐	ChIP-seq
☒	☐	Flow cytometry
☒	☐	MRI-based neuroimaging

Antibodies

Antibodies used	goat Anti GFP, ab6673, Abcam, diluted 1:2000; rabbit anti-RFP, 600-401-379, Rockland, diluted 1:1000, Donkey anti-goat-488, ab150129, Abcam, diluted 1:1000; Donkey anti-rabbit-594, R37119, ThermoFischer, diluted 1:1000
Validation	We used commercially and widely used antibodies to enhance GFP and RFP expression.

Animals and other research organisms

Policy information about studies involving animals; ARRIVE guidelines recommended for reporting animal research, and Sex and Gender in Research

Laboratory animals	For in vivo tracing (n=15, 8 males (m), 7 females (f)), ex vivo patch-clamp (n = 11, 7 m, 4 f), in vivo opto/electrophysiology (n = 6, 4 m, 2 f), in vivo vLGN terminal imaging (n=6, 4 m, 2 f), and in vivo TeLC experiments (n = 12, 6 m, 6 f), Gad2-IRES-Cre (JAX, #010802), aged 8 weeks (5–12 weeks for vLGN bouton imaging) at viral injection were used. For in vivo anterograde transsynaptic experiments, Ai75D (JAX, # 025106, n = 6, 4 m, 2 f), aged 8 weeks at viral injection were used. For in vivo retrograde transsynaptic experiments, NTSR1-GN209-Cre (MMRRC, #030780, n = 4, 2 m, 2 f), GRP-KH288-cre (MMRRC, #031183, n = 4, 2 m, 2 f), and Rorb-Cre (JAX, # 023526, n = 4, 2 m, 2 f), aged 8 weeks at viral injection were used. The mice for retinal terminal imaging experiments were C57BL/6J (JAX, #000664; n=5, 3 m, 2 f), aged 6–11 weeks at eye injection.
Wild animals	No wild animals have been used in the study
Reporting on sex	Female and male mice were used, showing similar results, see laboratory animals
Field-collected samples	No field-collected samples have been used in the study
Ethics oversight	All breeding and experimentation were performed under a license approved by the Austrian Federal Ministry of Science and Research in accordance with the Austrian and EU animal laws (BMF-66.018/0017-WF/V/3b/2017).

Note that full information on the approval of the study protocol must also be provided in the manuscript.

Plants

Seed stocks	N/A
Novel plant genotypes	N/A
Authentication	N/A
